# Autologous tumor-immune effusion cocultures enable ex vivo functional profiling of radiotherapy-immunotherapy combinations

**DOI:** 10.1186/s13046-026-03707-5

**Published:** 2026-04-14

**Authors:** Rebecca Zirnbauer, Daphni Ammon, Berta Mosleh, Nora Speiser, Anna Theophil, Markus Fabits, Mir Ali Reza Hoda, Michael Bergmann, Johannes Laengle

**Affiliations:** 1 Division of Visceral Surgery, Department of General Surgery, Comprehensive Cancer Center Vienna, Medical University of Vienna, Waehringer Guertel 18-20, Vienna, 1090 Austria; 2 Department of Thoracic Surgery, Comprehensive Cancer Center Vienna, Medical University Vienna, Waehringer Guertel 18-20, Vienna, 1090 Austria

**Keywords:** Pleural Effusion, Malignant, Ascites, Neoplasms, Tumor Microenvironment, Coculture Techniques, Radiotherapy, Immunotherapy, Immune Checkpoint Inhibitors, Precision Medicine, PATEC

## Abstract

**Background:**

Currently, radiotherapy-immunotherapy (RT-IO) combinations provide limited and heterogeneous benefits in human solid cancers and are frequently selected empirically, partly because human models that preserve native autologous tumor-immune interactions are lacking. We developed patient-derived autologous tumor-immune effusion cocultures (PATECs) as an ex vivo platform to functionally evaluate RT-IO regimens within an immunocompetent tumor microenvironment.

**Methods:**

Malignant pleural and peritoneal effusions (*n* = 29) from patients with metastatic solid cancers were processed for biobanking and primary tumor culture. Expandable tumor cultures were established in six effusions and recombined with matched autologous immune cells to generate PATEC. PATEC were treated with radiotherapy (RT), innate immune agonists (STING, TLR7/8), and immune checkpoint inhibitors (CTLA-4, PD-1, PD-L1, and TIGIT) in combinatorial regimens. Tumor cell death, T-cell activation, cytokine secretion, and CD8⁺ T-cell checkpoint expression were assessed using multiparametric flow cytometry and multiplex immunoassays. Contact dependence of cytotoxicity was evaluated by comparing tumor monocultures, direct cocultures, and transwell-separated cocultures.

**Results:**

Across conditions, regimens combining RT with a stimulator of interferon genes (STING) agonist were the most tumoricidal in PATEC, with marked interpatient variability and Bliss-defined synergy in a subset of effusions (3/6). STING agonist-mediated cytotoxicity required immune cells and was attenuated by the spatial separation of the tumor and immune compartments, whereas RT alone produced similar cytotoxicity in monocultures and cocultures at 72 h, suggesting that the observed RT effect in this assay was predominantly tumor-intrinsic. STING-based RT-IO induced early T-cell activation and a type I interferon-rich cytokine milieu, followed by increased expression of multiple inhibitory checkpoints on CD8⁺ T-cells. A composite CD8⁺ checkpoint co-expression score correlated with both overall and contact-dependent tumor cell death.

**Conclusions:**

PATEC enables the functional dissection of RT-IO combinations in a native effusion-derived tumor-immune microenvironment. The additional tumor cell killing conferred by STING-based RT-IO depends on immune cells and direct tumor-immune contact and varies between patient samples. These findings support the use of PATEC as a functional ex vivo system for testing therapeutic combinations in patient-specific settings.

**Supplementary Information:**

The online version contains supplementary material available at 10.1186/s13046-026-03707-5.

## Background

Advances in immunotherapy for solid cancers are perpetually evolving. The clinical use of immunomodulatory drugs to combat cancer has become more prominent over the last decade, with the number of patients eligible for immune checkpoint inhibition (ICI) increasing to 44% in 2018 [1]. However, only 12% of patients are estimated to respond to ICI therapy, and most indications involve monotherapy [1]. This has driven the inclination to combine immunomodulatory therapies. Within a couple of years several trials administering dual immunotherapy, including antibodies programmed death 1 (PD-1), programmed death ligand 1 (PD-L1), cytotoxic T lymphocyte associated protein 4 (CTLA-4), lymphocyte activation gene 3 (LAG-3) or T cell immunoreceptor with Ig and ITIM domains (TIGIT), as well as combinations with innate agonists targeting stimulator of interferon genes (STING) or Toll-like receptor 7/8 (TLR7/8), have been performed [2–6]. Moreover, preclinical studies have indicated that radiotherapy (RT) can act as an immunological adjuvant under specific biological and treatment conditions. Ionizing radiation can induce immunogenic tumor cell death, enhance tumor antigen release and major histocompatibility complex (MHC) class I expression, and activate cGAS-STING-mediated type I interferon (IFN-I) and inflammatory signaling within the tumor microenvironment (TME). These effects may collectively promote the recruitment and activation of effector lymphocytes and potentiate the efficacy of ICI and innate immune agonists [7–9]. Clinical trials that effectively combine RT with ICI in locally advanced solid tumors further support this rationale [10–14]. Notably, systematic analyses indicate that up to 72% of combinations lack an explicit biological rationale and that most combinations follow an empirical rather than a rational design [15, 16].

A rational approach to designing effective radiotherapy-immunotherapy (RT-IO) combinations is to identify them through functional testing in patient-derived systems. However, few approaches have been implemented, most likely because testing immunotherapies, particularly ICIs, requires scalable models that preserve a genuine autologous tumor immune microenvironment (TIME). Conventional immunocompetent mouse models and other standard preclinical models do not adequately recapitulate the complexity and interpatient heterogeneity of the human immune system in the context of the TIME. Precision-oriented platforms that use patient-derived materials offer the potential to gain insights into the local interactions of the TIME and design combination therapies with a translational rationale. Organotypic cultures derived from malignant effusions appear to offer a promising approach, allowing the expansion of effusion-derived tumor cells along with accessible autologous immune cells within a clinically relevant immunosuppressive TIME [17–19]. Published effusion-derived cultures have predominantly expanded tumor compartments for chemosensitivity and targeted therapy screening, whereas autologous immune compartments have not been preserved or functionally investigated. Furthermore, they do not maintain a native effusion TIME for systematic testing of immunotherapeutic combinations in autologous cocultures [17–20].

Malignant pleural and peritoneal effusions provide tumor cells, stromal fibroblasts, and autologous enriched tumor-associated immune cells that are naturally adapted to the liquid environment, making them feasible for ex vivo experiments. T cells, B cells, and myeloid cells are more similar to tumor-associated immune cells than peripheral blood mononuclear cells (PBMCs) in terms of their phenotypic and transcriptional states [21]. Effusion-derived T cells display increased expression of inhibitory checkpoints and exhaustion markers on T cells, enhanced transcriptional signatures for regulatory T cells, and macrophages have continuous M1/M2 phenotypes with more prominent M2 polarization characteristics [21–23]. Functional and TCR sequencing studies further indicated that malignant effusions harbor a clonally expanded memory phenotype and CD39^+^ CD8⁺ T cell subsets with features of tumor-reactive tumor-infiltrating lymphocytes, together with a cytokine and chemokine milieu enriched for vascular endothelial growth factor, interleukin-6, transforming growth factor-beta, and C-C motif chemokine ligand 22 that recapitulates key immunosuppressive attributes of the solid TIME [24–27]. Collectively, these phenotypic and functional similarities to solid TIME support the use of malignant effusions as a platform to investigate therapeutics that modulate the cancer-associated immune cell environment.

In this study, we developed a patient-derived autologous tumor-immune malignant effusion coculture (PATEC) model and used it to assess the functional effects of RT-IO combinations. We systematically tested combinations of innate agonists, as well as ICI in PATEC and identified the STING agonist, particularly in combination with RT, as the most potent inducer of tumor cell death in a subset of patients. STING agonist-mediated cytotoxicity is largely immune cell-dependent and requires direct immune-tumor contact. By condensing the distribution of multi-checkpoint-positive CD8⁺ T cell phenotypes into a composite checkpoint co-expression score (CES), we linked these checkpoint co-expression states specifically to the magnitude of overall and contact-dependent tumor cell killing. Overall, these data provide proof‑of‑concept that malignant effusion-derived PATECs can serve as a patient‑specific ex vivo platform to functionally test and characterize RT-IO regimens within an autologous TIME.

## Methods

### Acquisition and processing of malignant effusions

Malignant effusion samples (ascites and pleural) were prospectively collected from patients with cancer undergoing drainage procedures performed by the Departments of Thoracic Surgery and General Surgery. Only pathologically confirmed malignant effusions (Department of Pathology) were included. All patients provided written informed consent. The study was approved by the local Ethics Committee (EC) of the Medical University of Vienna (No. 2042/2019). Samples were collected aseptically in sterile containers or fluid bags (1–2 L) and transported to the laboratory. Samples were processed within 3 h of collection under aseptic conditions in a laminar flow cabinet and filtered through a 100 μm cell strainer (pluriStrainer^®^, pluriSelect) into 50 mL conical Falcon tubes (Sarstedt AG & Co.) to remove fibrotic tissue and aggregates. After multiple washes with phosphate-buffered saline (PBS; Gibco) and centrifugation steps at 400×rcf for 10 min, erythrocytes were lysed using ACK lysis buffer (Gibco™ A1049201). Key reagents used for sample processing and cell culture are summarized in Additional file 1: Table S1A. Cell counts were assessed using an automated Sysmex Cell Counter XN 350 (Sysmex). Samples with < 100 × 10^6^ nucleated cells were excluded from the cultivation of primary tumor cell cultures. Cells were resuspended in CryoStor^®^ CS10 medium (MKCL1984, C2874) in addition to 10 µM Y-27,632 dihydrochloride (MedChemExpress, HY 10071/CS 0131) and aliquoted into sterile 2 mL cryotubes (VWR), and gradually frozen overnight using CoolCell LX containers (BioCision) at -80 °C before transfer to liquid nitrogen storage until further use.

### Development and characterization of primary tumor cell cultures

Primary tumor cell cultures (PTCCs) were developed by seeding CD45-depleted cells into standard adherent culture flasks (25 cm²) using Advanced DMEM/F12 medium (Gibco, Cat#12634010) with 10% fetal bovine serum (FBS, Thermo Fisher, Cat#10500064), 1% antibiotic antimycotic solution (Sigma, Cat#A5955), and 10 µM Y-27,632 dihydrochloride. Cryopreserved malignant effusion-derived cells were rapidly thawed in 100% FBS supplemented with 10 µM Y-27,632, washed once with protocol medium, and centrifuged at 400×rcf for 5 min. Leukocyte depletion was performed using the EasySep™ Human CD45 Depletion Kit (Stemcell Technologies, Cat#17898) according to the manufacturer’s protocol, using 5 mL polystyrene round-bottom tubes (Stemcell Technologies, Cat#38007) and the EasySep magnetic cell isolation system. Tumor-enriched cells were cultured at 37 °C in a humidified incubator with 5% CO₂, with medium changes every 2–3 days. Successful primary tumor culture development was defined as growth and proliferation beyond three passages (> P3).

### Immunohistochemical (IHC) analysis of primary tumor cell cultures

Primary tumor cells seeded onto gelatin-coated slides were fixed in 2% formaldehyde for 20 min at room temperature after confluence. Antigen retrieval was performed using citrate buffer in an autoclave at 121 °C. Endogenous peroxidase was blocked (BLOXALL™, Vector Laboratories) for 10 min. Following blocking with 10% horse serum, slides were incubated for 60–90 min at room temperature with primary antibodies diluted in 10% horse serum: EpCAM (clone HEA 125, MACS Miltenyi, 1:80), pan-cytokeratin (PanCK) (clone C11, Cell Signaling, 1:500), and STING (clone D2P2F, Cell Signaling, 1:400). Details of the antibodies used for immunohistochemistry are provided in Additional file 1: Table S1B. Detection was performed using ImmPRESS™ Excel Polymer Reagent and visualization was performed using a DAB substrate (Vector Laboratories). Slides were counterstained with hematoxylin and mounted using Entellan™ (Merck). Negative controls without primary antibody were included. Imaging was performed using bright-field microscopy with a Vectra Polaris™(Akoya Biosciences).

### Flow Cytometry (FCM) analysis of cellular composition and checkpoint receptor analysis

Cells isolated from malignant effusions and primary tumor cultures were characterized using multiplex flow cytometry. Following thawing and washing, Fc receptor blockade was performed using human AB serum (diluted 1:1 with PBS containing 2% FBS) at 4 °C. Cells were then stained with Zombie Violet™ or Zombie Yellow™ viability dye (BioLegend) and incubated with fluorochrome-conjugated monoclonal antibodies targeting CD45 (AF700, BioLegend), EpCAM (AF488, eBioscience), CD3 (FITC, BioLegend), CD4 (APC, BioLegend), CD8a (PE, BioLegend; AF700, BioLegend), CD56 (PE, BioLegend), CD14 (PE-Cy7, BioLegend), CD11c (PerCP-eFluor710, eBioscience), HLA-DR (APC-Cy7, BioLegend), CD20 (APC, BioLegend), CD163 (PerCP-eFluor710, eBioscience), CD66b (PE, BioLegend), CD11b (APC-Cy7, BioLegend), and CD86 (BV650, BioLegend). For checkpoint receptor analysis, cells were stained with antibodies specific to CTLA-4 (PE-Dazzle 594, BioLegend), TIGIT (PerCP-eFluor710, eBioscience), LAG3 (PE-Cy7, BioLegend), PD-1 (BV510, BioLegend), PD-L1 (BV785, BioLegend), and TIM3 (APC-Cy7, BioLegend). A complete list of fluorochrome-conjugated antibodies used for immune phenotyping and checkpoint receptor analysis is provided in Additional file 1: Table S1C. Corresponding IgG isotype controls matched for each fluorochrome were included. Details of the isotype control antibodies are provided in Additional file 1: Table S1D. Compensation was performed using UltraComp eBeads™ (Invitrogen). Data were acquired on a CytoFLEX flow cytometer (Beckman Coulter) and analyzed using Kaluza Analysis 2.1 (Beckman Coulter).

### Patient-derived Autologous Tumor-immune Effusion Coculture (PATEC) experiments

For the PATEC coculture assays, primary tumor cells and matched autologous immune cells were combined at a fixed tumor-immune ratio of 1:5 (5 × 10⁴ tumor cells and 2.5 × 10⁵ immune cells per well) in flat-bottom 24-well plates (Greiner Bio One). This standardized ratio was selected to enable a controlled comparison of treatment-induced tumor cell death across samples with markedly variable baseline effusion composition and frequent leukocyte predominance. Similar 1:5 target: effector conditions have been used in prior tumor-immune coculture studies, and published work indicates that the target: effector ratio substantially influences the extent of measured killing and nonspecific target loss [28–30]. Accordingly, tumor cell input was kept constant and matched immune cells were added in moderate excess for all tumor-killing assays. The cells were cultured in DMEM/F12 medium (Gibco) in addition to 10% heat inactivated fetal bovine serum (FBS, Thermo Fisher) and 1% Antibiotic Antimycotic Solution (Sigma) in a humidified incubator at 37 °C with 5% CO₂. After seeding tumor cells, they were incubated overnight prior to immune cell addition. Selected cultures were subjected to radiotherapy (8 Gy; YXLON Maxishot X-ray system, 200 kV, ~ 1 Gy/min). Immunotherapeutic treatments included STING agonist ADU-S100 (10 µM, InvivoGen), TLR7/8 agonist Resiquimod (R848; 10 µg/mL, InvivoGen), and checkpoint inhibitors ipilimumab (anti CTLA-4; Yervoy, 10 µg/mL), atezolizumab (anti PD-L1; Tecentriq, 10 µg/mL), pembrolizumab (anti PD-1; Keytruda, 10 µg/mL), and tiragolumab (anti-TIGIT; 10 µg/mL, Selleck Chemicals, Cat#A2028), alone or in combinations as indicated. After 72 h, the cells were detached using Accutase (Sigma-Aldrich) for flow cytometric analysis. Experiments were performed in duplicate.

### Spatial separation of PATEC

To determine cell contact dependence, tumor cells and immune cells were cultured in direct contact or spatially separated using ThinCert well inserts with a pore size of 0.4 μm (Greiner Bio One, 662640). Tumor cells were seeded at 5 × 10^4^ cells per well in 24-well plates. For the insert-separated PATEC, 2.5 × 10^5^ matched autologous immune cells were seeded per insert, corresponding to the same fixed tumor-immune ratio of 1:5 used in the direct PATEC coculture experiments. Immunotherapeutic treatment with the STING agonist (ADU-S100, 10 µM) and RT (8 Gy) was applied as described above. After 72 h of incubation at 37 °C and 5% CO₂, cells were harvested using Accutase, stained with fluorochrome-conjugated monoclonal antibodies, and analyzed using FCM.

### FCM analysis of treatment outcomes: tumor cell death and immune checkpoint receptor expression

Tumor cell death was quantified after 72 h of incubation post-treatment by FCM using Zombie Violet™ viability dye (BioLegend) and Calcein AM (BioLegend). Positive controls were generated through repeated freeze-thaw cycles. Viable (Calcein AM⁺/Zombie Violet⁻) and dead (Zombie Violet⁺) cell populations were quantified, and the results were expressed as fold changes relative to untreated controls. Additionally, immune checkpoint receptor expression was analyzed on CD8⁺ and CD4⁺ T cells.

### Assessment of T cell activation

Immune cells isolated from malignant effusions were thawed and resuspended in assay medium (DMEM/F12, 10% FBS, antibiotic antimycotic solution). Cells were seeded at 1 × 10^5^ cells per well into 96-well U bottom plates (Greiner Bio One) and treated with STING agonist ADU-S100 (10 µM, InvivoGen) and/or RT (8 Gy; YXLON Maxishot, 200 kV X-ray, ~ 1 Gy/min). Following 24 h of incubation at 37 °C and 5% CO₂, cells were stained with fluorochrome-conjugated antibodies against CD3 (FITC, BioLegend), CD4 (APC, BioLegend), CD8 (PE, BioLegend), CD45 (eFluor450, Invitrogen), CD69 (PerCP-eFluor 710, eBioscience), and CD107a (PE-Cy7, BioLegend). Compensation controls were prepared using UltraComp eBeads™ (Invitrogen). Samples were acquired on a DxFLEX flow cytometer (Beckman Coulter), and expression levels of activation markers CD69 and CD107a on CD4⁺ and CD8⁺ T cells were analyzed using Kaluza software (Beckman Coulter).

### Multiplex cytokine profiling of culture supernatants

The culture supernatants were harvested 24 h post-treatment and analyzed for cytokine secretion using the Luminex^®^ Human Discovery Assay (13-Plex) LXSAHM-13 (Bio-Techne Ireland Limited) following the manufacturer’s protocol. The cytokine panel included TNF-α, IL-6, IP-10, IFN-α, MIP-1α, MIP-1β, MCP-1, IL-1ra, IL-8, RANTES, IFN-β, IFN-γ, and IL-10. Data acquisition was performed using a Luminex^®^ FlexMap 3D, and analyses were conducted using xPONENT^®^ 4.3 analysis software (Luminex^®^).

### Statistical analysis

Statistical analyses were performed in R (version 2025.05.1 + 513) using the lme4 and lmerTest packages for linear mixed‑effects models, emmeans or multcomp for multiple comparisons, and ggplot2 together with dplyr and tidyr for data handling and visualization. Treatment effects across multiple patients were evaluated using linear mixed-effects models, adjusting for patient-specific random effects. Post hoc pairwise comparisons between treatment groups were conducted using Tukey’s multiple comparisons test. Cytokine concentrations were normalized using z-score transformation for comparative analyses. Additionally, intra-patient analyses were performed using one-way ANOVA followed by Tukey’s post hoc test, and, where appropriate, by two-way ANOVA with factors Treatment and Culture condition followed by Tukey’s post hoc test. For checkpoint expression panels with multiple CD8⁺ subsets, family-wise multiplicity across the panel was controlled (Holm or Benjamini-Hochberg false discovery rate) in addition to the within-panel Dunnett adjustments. For cytokines, p-values were adjusted within the cytokine family using the Benjamini-Hochberg FDR. Statistical significance was defined as follows: *p < 0.05, **p < 0.01, ***p < 0.001, and ****p < 0.0001.

CD8⁺ checkpoint panels and CES-killing analyses were conducted on the logit scale. Percent positive values were logit-transformed and analyzed using Gaussian linear mixed-effects models with condition as a fixed effect and patient as a random intercept; a random slope for condition was included when supported by the split-plot design. For display, values are shown as fold change versus the patient matched control, while inference used the logit model. Omnibus p-values for condition derive from the mixed model; multiplicity-adjusted post hoc comparisons versus control were obtained using Dunnett contrasts. The CD8⁺ checkpoint co-expression score (CES; day 3, within PATEC) was computed by z-scoring the % gated frequencies of PD-1⁺LAG-3⁺TIM-3⁺, PD-1⁺TIGIT⁺LAG-3⁺, PD-1⁺TIGIT⁺TIM-3⁺, and PD-1⁺TIGIT⁺LAG-3⁺TIM-3⁺ and combining as CES = mean(z of the three triple positive subsets) + 2×z(quadruple positive). Tumor cell death at 72 h was summarized as Δlogit = logit(%dead_treated) – logit(%dead_control), and the contact-dependent component as Δ(contact) = Δlogit(Direct) − Δlogit(Insert). Associations with CES were evaluated by Gaussian LMMs with patient random intercepts (Δlogit ~ CES + (1|patient); Δ(contact) ~ CES + (1|patient)); fixed effect slopes (β) are reported in log odds per CES unit with Wald 95% confidence intervals, Satterthwaite P values (lmerTest), and Nakagawa R² (marginal; conditional). For Δ(contact), the random intercept variance was ≈ 0 (singular fit), hence R²_cond ≈ R²_marg; improvement over a null (intercept only) model was checked by the likelihood ratio test, and leave-one-PATEC-out refits were used as a stability check.

Synergy between STING agonist and RT was quantified using the Bliss independence model applied to control-normalized fractional killing. For each patient and experiment, the percentage of Zombie Violet™-positive tumor cells in untreated controls (d_ctrl) and in treated wells (d) was converted to fractions and expressed as additional killing on the surviving population, $\:\boldsymbol{E}=\frac{\boldsymbol{d}-{\boldsymbol{d}}_{\mathrm{ctrl}}}{1-{\boldsymbol{d}}_{\mathrm{ctrl}}}$, truncated to the interval [0,1]. Bliss expectation was calculated as $\:({\boldsymbol{E}}_{\mathrm{bliss}}={\boldsymbol{E}}_{\mathrm{STING}}+{\boldsymbol{E}}_{\mathrm{RT}}-{\boldsymbol{E}}_{\mathrm{STING}}\times\:{\boldsymbol{E}}_{\mathrm{RT}})$. The Bliss synergy index is defined as $\:\boldsymbol{\Delta\:}\boldsymbol{E}={\boldsymbol{E}}_{\mathrm{obs}}-{\boldsymbol{E}}_{\mathrm{bliss}}$ ​, expressed as percentage points. For each patient, replicate level $\:\boldsymbol{\Delta\:}\boldsymbol{E}$ values were summarized as the mean with 2,000-sample non-parametric bootstrap 95% confidence intervals; combinations were classified as synergistic when the entire confidence interval lay above + 5% points and otherwise as neutral.

## Results

### Development and characterization of Patient-derived Autologous Tumor-immune Effusion Cocultures (PATEC)

**Fig. 1:**
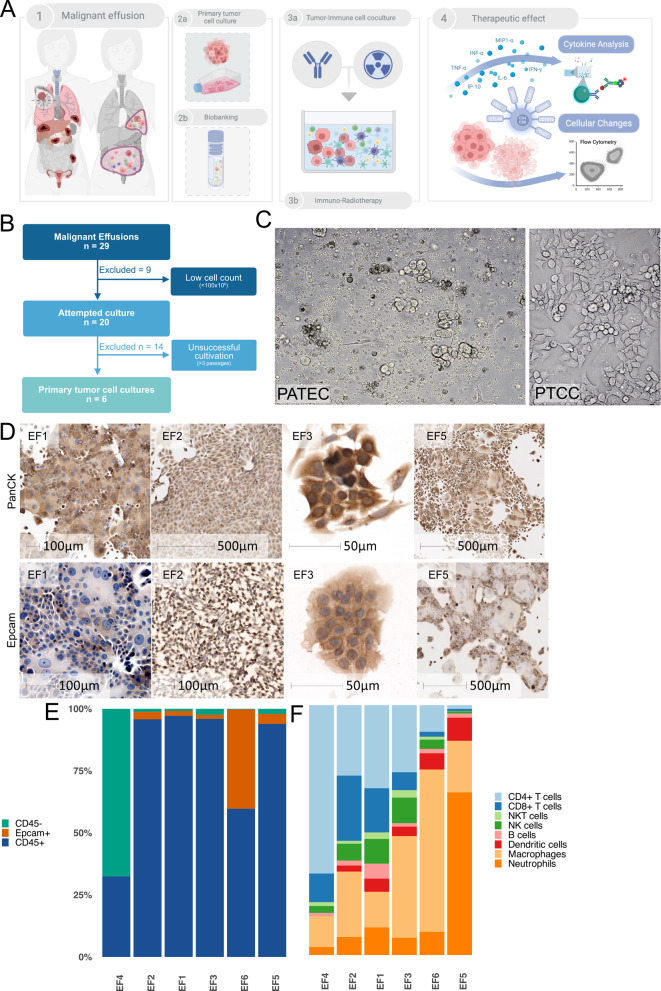
Development of primary tumor cell cultures (PTCCs) and patient-derived autologous tumor-immune effusion cocultures (PATEC). **A** Schematic overview of the experimental workflow. **B** CONSORT diagram summarizing PTCC development from malignant effusions. **C** Representative bright-field images of PATEC and PTCC. **D** Immunohistochemical characterization of PTCCs. Representative images show EpCAM and PanCK positivity (brown chromogen) with hematoxylin counterstaining (blue). Scale bars as indicated. **E** Cellular composition of malignant effusions (EF1-EF6, *n* = 6) quantified by flow cytometry, showing the proportions of CD45⁺ leukocytes, EpCAM⁺ epithelial tumor cells, and CD45⁻EpCAM⁻ non-hematopoietic cells. **F** Relative abundances of immune subsets within the CD45⁺ compartment

We hypothesized that malignant effusions, which contain both tumor and immune cells within their native fluid microenvironment, could serve as a source for generating autologous cocultures to study RT-IO responses. To test this, malignant pleural effusions (*n* = 23) and ascites (*n* = 6) were prospectively collected and processed for biobanking and primary tumor cell culture (Fig. 1A). Nine samples were excluded due to insufficient nucleated cell count (< 100 × 10⁶ cells). Of the 20 cultures attempted, six maintained continuous proliferation beyond three passages and were expanded as primary tumor cell cultures (PTCCs) for downstream functional coculture assays (Fig. 1B). Clinical and sample characteristics of the acquisition cohort are summarized in Table 1 (Additional file 1: Table S2). Among cultures attempted (*n* = 20), long-term PTCC establishment (> P3) was observed in pancreatic (3/4; 75.0%), ovarian (1/4; 25.0%), lung (1/6; 16.7%), and CUP (1/1; 100.0%) cases, but was not observed in gastric (0/3), breast (0/1), or colorectal (0/1) cases (Additional file 1: Table S3A). In exploratory univariable analyses restricted to attempted cultures, establishment occurred more frequently in stage IV disease at diagnosis than in stage I–III disease at diagnosis (6/12 (50.0%) vs. 0/8 (0.0%), *p* = 0.042), while ascites and pancreatic primaries showed numerically higher establishment than pleural effusions and non-pancreatic primaries (each 3/4 (75.0%) vs. 3/16 (18.8%), *p* = 0.061) (Additional file 1: Table S3B).

**Table 1:** Clinical and sample characteristics of malignant effusions (*n* = 29)

Characteristic	
Effusion site, No. (%)	
Pleural effusion	23 (79)
Ascites	6 (21)
Primary tumor, No. (%)	
Lung	7 (24)
Pancreas	6 (21)
Ovary	5 (17)
Breast	4 (14)
Gastric	3 (10)
Colorectal	2 (7)
Appendix	1 (3)
CUP	1 (3)
Histologic subtype, No. (%)	
Adenocarcinoma	19 (65)
Serous ovarian carcinoma	5 (17)
Breast carcinoma	4 (14)
Neuroendocrine carcinoma	1 (3)
Stage at diagnosis, No. (%)	
I	4 (14)
II	3 (10)
III	8 (28)
IV	14 (48)
Metastatic disease at effusion sampling, No. (%)	29 (100)
Tumor grade, No. (%)	
G1	2 (7)
G2	6 (21)
G3	17 (59)
Unknown	4 (14)
Sex, No. (%)	
Female	17 (59)
Male	12 (41)
Age, median (range)	62 (37–81)
Pre-sampling anticancer treatment exposure, No. (%)	
Radiotherapy prior to effusion sampling	11 (38)
Any systemic therapy prior to effusion sampling	22 (76)
Chemotherapy	21 (72)
Targeted therapy	15 (52)
Immune checkpoint inhibitor therapy	4 (14)
Endocrine therapy	2 (7)
Total nucleated cell yield at processing (×10⁶), median (IQR)	300 (94–750)
Culture attempted, No. (%)	20 (69)
Long-term PTCC established among all samples, No. (%)	6 (21)
Long-term PTCC established of attempted cultures, No. (%)	6/20 (30)

Categories for systemic therapy modality are not mutually exclusive. *IQR* interquartile range, *CUP* cancer of unknown primary, *PTCC* primary tumor cell culture, *PATEC* patient-derived autologous tumor–immune effusion coculture, *ICI* immune checkpoint inhibitor. Culture attempted indicates PTCC cultivation only if the total nucleated cell yield at processing was ≥ 100 × 10⁶ cells (Sysmex); samples with yields below this threshold were excluded. Long-term PTCC established denotes proliferation beyond three passages

### Immunohistochemical validation of Primary Tumor Cell Cultures (PTCCs)

Bright-field microscopy demonstrated PTCCs as adherent polygonal epithelioid cells forming cohesive nests and multilayered aggregates with peripheral cell detachment. In PATEC, numerous small mononuclear cells were visible in the foreground, with interspersed tumor cell aggregates observed in suspension, reflecting the mixed tumor-immune composition of the coculture (Fig. 1C). To confirm epithelial tumor origin, immunohistochemistry (IHC) staining of PTCCs was performed. PTCCs displayed strong cytoplasmic expression of PanCK and membranous expression of EpCAM in adherent cells, confirming epithelial origin (Fig. 1D). FCM analysis within the CD45⁻ compartment showed tumor cells expressed the epithelial marker EpCAM in five of six cases, consistent with the underlying tumor histology, while one sample (EF4), derived from a dedifferentiated lung adenocarcinoma, lacked EpCAM expression (Fig. 1E).

### Heterogeneous immune cell composition of malignant effusions

FCM demonstrated pronounced heterogeneity in the cellular composition of malignant effusions (Fig. 1E-F, Additional file 2: Fig. S1). Leukocytes (CD45⁺) were the dominant population in most samples, making up over 90% of cells. The immune compartment showed patient-specific variation, with macrophages and neutrophils as the most abundant subsets in three of six effusions, and variable proportions of CD4⁺ and CD8⁺ T cells, NK cells, NKT cells, dendritic cells, and B cells. This interpatient diversity reflects the individualized immune and tumor composition of malignant effusions and provides a genuine TIME, forming a basis for patient-specific functional testing of immunomodulatory drugs. Collectively, these results establish a workflow to generate PTCCs that can be paired with autologous immune compartments, enabling downstream use of PATEC for functional RT-IO testing. With scalable patient-matched tumor-immune cells from one compartment, we next assessed therapeutic efficacy across immunomodulatory combinations to determine the most efficacious treatment regimen.

### Combinatorial RT-IO screening in PATEC identifies STING agonist and RT as an effective therapeutic combination

**Fig. 2:**
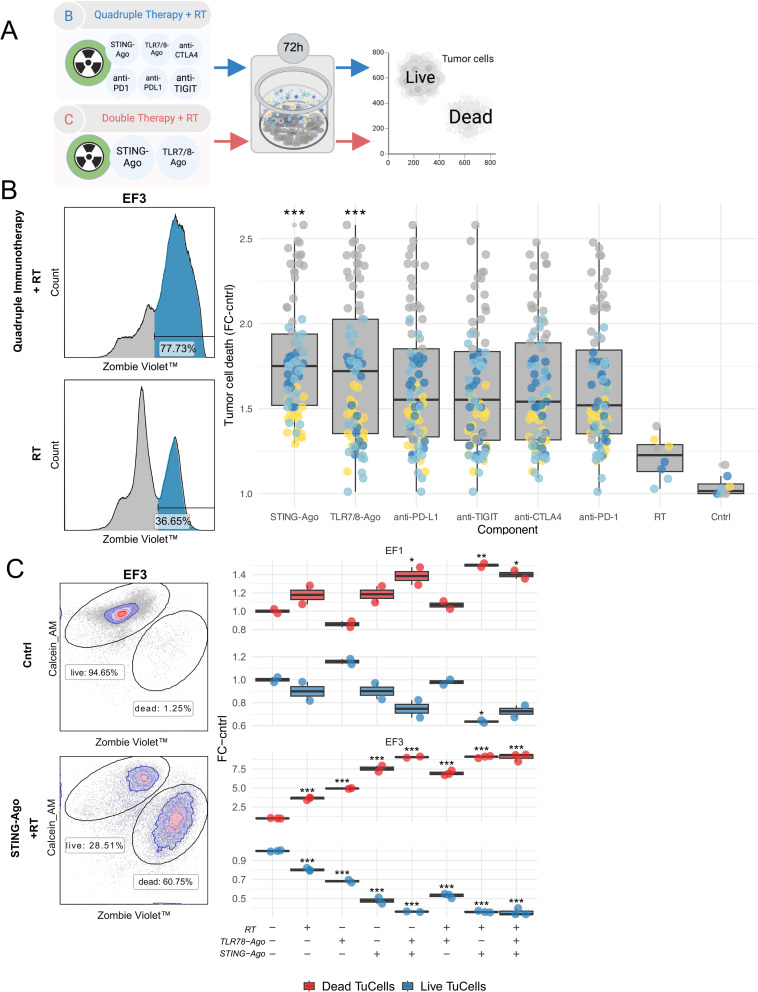
Combinatorial RT-IO screening in PATEC identifies STING agonist and RT as an effective therapeutic combination. **A** Experimental schematic. **B** Quadruple RT-IO combinations increase tumor cell death in PATEC. Representative histograms (left) depict tumor cell death measured by flow cytometry at 72 h culture after treatment. Boxplots (right) display the fold change in % of tumor cell death relative to control (cntrl; *n* = 4). Each boxplot summarizes all quadruple immunotherapy combinations containing the indicated immunotherapeutic agent, with RT (8 Gy) included in all. Colored dots represent technical replicates from PATEC (dark blue = EF1, yellow = EF2, gray = EF3, light blue = EF4). Median, IQR, whiskers 1.5×IQR. LMM; patient random intercept; Holm. **C** Tumor cell death in PATECs (EF1 and EF3, *n* = 2) after 72 h of RT, STING agonist, TLR7/8-Ago, and their combinations. Representative flow cytometric density plots (left). Boxplots summarize tumor cell death, quantified as fold change relative to control (Δ Control, *n* = 2). Dots indicate technical replicates. One‑way ANOVA; Tukey multiple comparisons. *p < 0.05; **p < 0.01; ***p < 0.001

To functionally assess the capacity of the PATEC platform to capture immune-mediated tumor cytotoxicity, we performed an initial combinatorial screen integrating innate and adaptive immunotherapies with RT (8 Gy) (Fig. 2A). Quadruple RT-IO included two innate agonists targeting STING and TLR7/8 together with ICI PD-1, PD-L1, CTLA-4, and TIGIT. Tumor cell death was quantified at 72 h by FCM using Zombie Violet™ and Calcein AM. Exemplary histograms illustrate a marked shift towards Zombie Violet⁺ cells following treatment compared with RT alone (Fig. 2B, left). Across four independent PATECs, quadruple RT-IO that included an innate agonist (STING agonist or TLR7/8 agonist) together with RT significantly increased tumor cell death compared with RT alone or untreated controls (Fig. 2B, right; component-wise linear mixed-effects model with patient random intercept; Holm adjustment; *p* < 0.001, Additional file 2: Fig. S2A-S2C). This demonstrates that PATEC can be used as an ex vivo human system capable of detecting immune-mediated tumor killing under relevant combination regimens.

### Combinations with STING agonist contributed most strongly to tumor killing

To delineate which innate immune modulatory component contributed to the observed response, we compared STING agonist and TLR7/8-Ago, alone or in combination with RT, in representative PATECs (EF1 and EF3). Tumor cell death, expressed as fold change relative to matched controls, increased with either agonist, however the STING agonist + RT combination produced the most pronounced killing across replicates (one-way ANOVA with Tukey multiple comparisons; *p* < 0.05, Fig. 2C, Additional file 2: Fig. S2D). STING agonist monotherapy also induced measurable cytotoxicity in the selected PATECs, whereas TLR7/8 agonism alone or with RT had more modest effects. Notably, RT potentiated STING agonist efficacy, suggesting cooperative mechanisms between DNA damage signaling and STING activation. Collectively, these findings indicate that STING agonism in combination with RT is the leading combination for inducing tumor cell death in these selected PATECs. We then investigated interpatient variability and potential synergy between STING agonism and RT across PATECs.

### Patient-specific variability in response to STING agonist and RT in PATECs

**Fig. 3:**
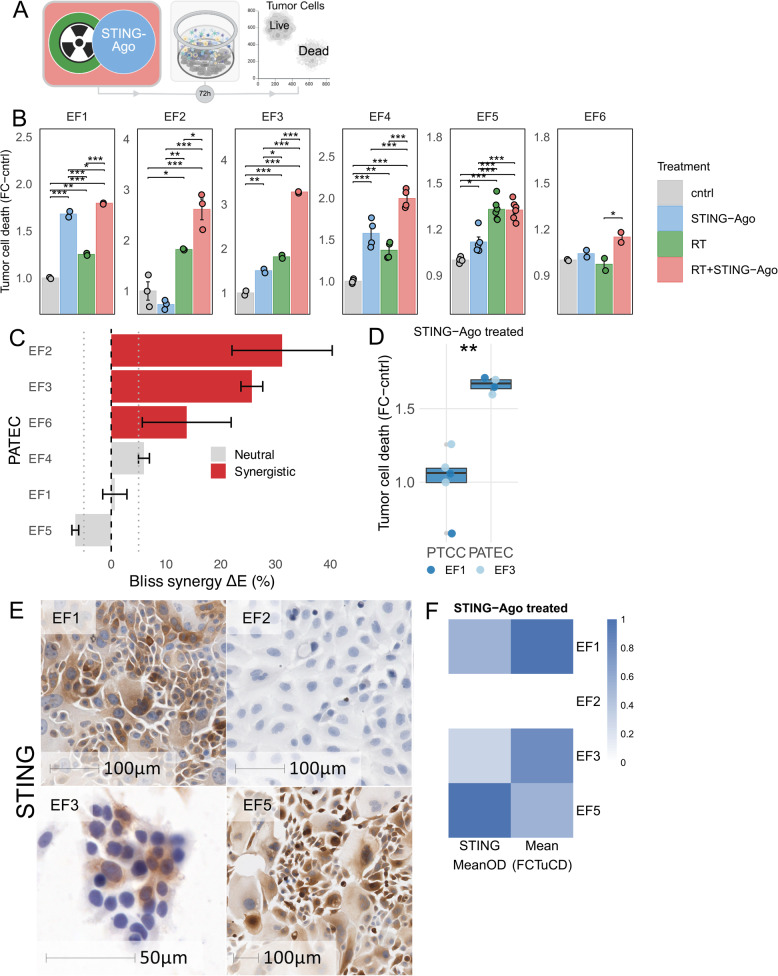
Patient‑specific tumor cell killing and synergy of STING agonist and RT in PATECs. **A** Experimental schematic. **B** Tumor cell death in PATECs (EF1-EF6, *n* = 6) after 72 h of treatment. **C** Bliss synergy index (ΔE, %) for RT + STING agonist for PATECs (EF1-EF6), derived from control-normalized fractional tumor cell death. Bars show mean ΔE with 95% bootstrap confidence intervals; dashed and dotted lines indicate ΔE = 0 and the ± 5% reference bounds. **D** Comparison of tumor cell death after 72 h of STING agonist treatment in primary tumor monocultures (PTCC) versus matched autologous cocultures (PATEC, *n* = 2). **E** Representative IHC images of formalin-fixed primary tumor cell cultures stained for STING (brown chromogen) and hematoxylin (blue). **F** Heatmap of mean cytoplasmic STING staining intensity (optical density) in PTCCs and the corresponding STING agonist–induced fold change in tumor cell death at 72 h in the matched PATECs (*n* = 4 patients). Treatments: STING agonist (10 µM ADU-S100), RT (8 Gy), RT + STING agonist (8 Gy + 10 µM ADU-S100). **B**,** D**,** F** Fold change of % Zombie Violet™⁺ tumor cells relative to control (cntrl). **B** Bars represent the mean ± s.e.m. of technical replicates. Two-way ANOVA; Tukey’s multiple comparisons. **C** Bars show the mean ΔE with 95% bootstrap confidence intervals (2,000 resamples). **D** Boxes show the median and IQR; whiskers 1.5×IQR; dots are technical replicates. LMM; emmeans pairwise contrast PTCC vs. PATEC. **E**,** F** descriptive only; no additional statistical testing beyond that specified in the text and Methods. *p < 0.05; **p < 0.01; ***p < 0.001

To assess the patient-specific efficacy of STING agonism and RT, six PATECs (EF1-EF6) were treated with the STING agonist, RT, or the combination for 72 h (Fig. 3A). In five of six PATECs, combination therapy showed the most pronounced tumor cell death, whereas in EF5 the effect of RT + STING agonist was comparable to RT alone. The magnitude of response varied markedly between PATECs. EF2 and EF3 exhibited the most pronounced cytotoxicity (two- to three-fold), while EF1 and EF6 displayed modest increases. This variability was observed both across tumor types and within pancreatic adenocarcinoma, in which the three PATECs showed divergent 72 h response magnitudes (Additional file 1: Table S4). Two-way ANOVA with Tukey’s multiple comparisons confirmed significant treatment effects in all PATECs (*p* < 0.05, Fig. 3B, Additional file 2: Fig. S3A). Together, these data show that STING agonism, particularly in conjunction with RT, can increase tumor cell death in PATECs, but with marked interpatient variability in response magnitude.

### A subset of PATECs exhibits synergistic STING-RT interactions

Next, we examined whether combining STING agonism and RT resulted in greater-than-additive cytotoxicity. Bliss independence modelling identified synergy in a subset of PATECs (Fig. 3C). EF2, EF3, and EF6 showed positive Bliss indices ( > + 5%) with 95% CIs (2,000 bootstrap resamples) above + 5%, indicating synergy, whereas EF1, EF4, EF5 did not. Bliss-defined synergy was observed across more than one tumor type and was not uniformly present within pancreatic adenocarcinoma, which comprised one synergistic and two neutral PATECs (Additional file 1: Table S4). This pattern is consistent with functional heterogeneity in responsiveness to STING agonism and RT within PATECs.

### STING agonist-induced cytotoxicity in EF1 and EF3 depends on immune cells

We compared primary tumor monocultures (PTCC) with matched autologous tumor-immune cocultures (PATEC) to assess whether the cytotoxic effects of STING activation are immune cell-dependent. In the two matched pairs analyzed (EF1 and EF3), tumor cell death after 72 h of STING agonist treatment was higher in PATEC than in matched PTCC (Fig. 3D, Additional file 2: Fig. S3B), suggesting that STING-mediated cytotoxicity may depend on the immune cell compartment in these samples.

### STING expression in tumor cells varies across patients

IHC staining of primary tumor cell cultures (PTCCs) showed heterogeneous cytoplasmic STING expression across four patients (EF1, EF2, EF3 and EF5) (Fig. 3E-F). Representative images illustrate strong cytoplasmic staining in EF1 and EF5, whereas EF2 and EF3 were weak or negative (Fig. 3E). A descriptive comparison of mean optical density (OD) and corresponding fold change in tumor cell death after STING agonist exposure suggested that higher baseline STING expression may coincide with increased responsiveness in some samples (Fig. 3F). However, the limited cohort precludes definitive correlation.

**Fig. 4:**
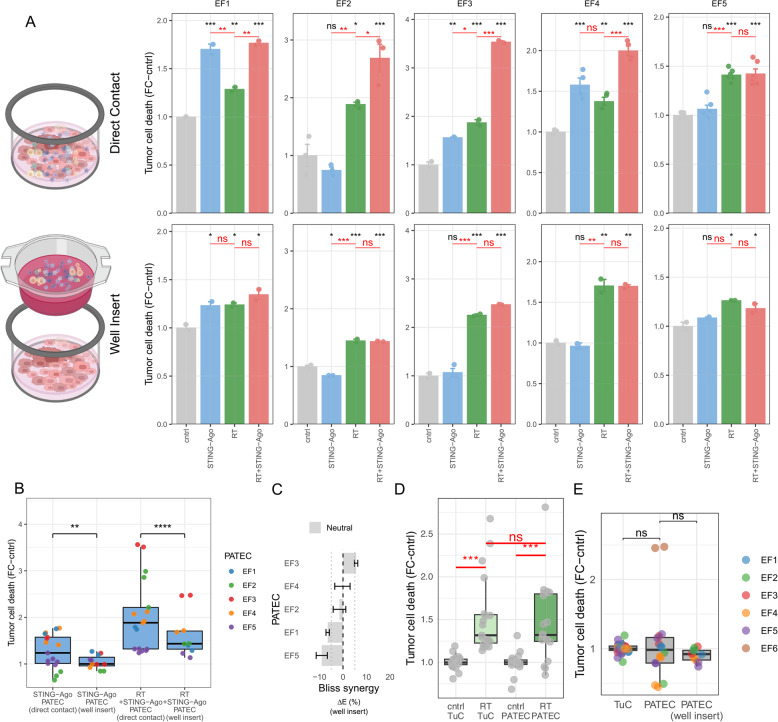
Effect of STING agonist and RT on cytotoxicity in PATEC depends on immune cell contact. **A** Tumor cell death in PATEC (*n* = 5) after 72 h in direct coculture with immune cells or in insert-separated coculture treated with STING agonist, RT, or RT + STING agonist. Comparisons with cntrl (black) and between treatments (red) are shown above the bars. **B** Direct versus insert-separated conditions for STING agonist and RT + STING agonist at 72 h (*n* = 5). **C** Bliss synergy index (ΔE, percentage points) for RT + STING agonist for insert-separated condition, summarized per PATEC (EF1-EF5) as mean ΔE with 95% bootstrap confidence intervals; dashed and dotted lines indicate ΔE = 0 and the ± 5% neutrality band. **D** Tumor cell death at 72 h in TuC and PATEC after RT or cntrl (*n* = 5). **E** Tumor cell death in untreated TuC, direct PATEC, and insert-separated PATEC (well insert) at 72 h (*n* = 6); ns, not significant. Treatments: STING agonist (10 µM ADU-S100), RT (8 Gy), RT + STING agonist (8 Gy + 10 µM ADU-S100). **A**,**B**,**D**,**E** Fold change of % Zombie Violet™+ cells relative to cntrl. **A** Bars represent mean ± s.e.m. of 2–4 technical replicates (dots). Two-way ANOVA; Tukey’s multiple comparisons. **B**,**D**,** E** Boxes show the median and IQR; whiskers 1.5×IQR. LMM; patient random intercept; Tukey-adjusted pairwise contrasts. ****p < 0.0001, ***p ≤ 0.001, **p < 0.01, *p < 0.05; ns, not significant

These data show variable but reproducible STING agonist-induced tumor cell killing in the analyzed PATECs and synergistic effects with RT in a subset of samples. In EF1 and EF3, STING-mediated cytotoxicity was evident only in the coculture setting, supporting dependence on the immune cell compartment. Therefore, we next examined whether the observed cytotoxicity required direct immune-tumor contact (Fig. 4).

### Direct immune-tumor contact is critical for the cytotoxic efficacy of STING agonist-based RT-IO

Next, we examined whether physical proximity between tumor and immune cells is necessary for the cytotoxic effects of STING agonist-based RT-IO. PATECs were treated for 72 h with the STING agonist, RT, or the combination, either in direct coculture or separated by a transwell insert (Fig. 4A, Additional file 2: Fig. S4A). Across five independent PATECs, RT + STING agonist elicited a marked increase in tumor cell death under direct contact conditions, whereas spatial separation abolished this response (two-way ANOVA with Tukey’s multiple comparisons; *p* < 0.05). Pooled analysis across PATECs confirmed significantly higher killing for both STING agonist alone and RT + STING agonist in direct contact compared with insert-separated PATEC (linear mixed-effects model with patient random intercept; *p* < 0.01, *p* < 0.001; Fig. 4B, Additional file 2: Fig. S4B). Consistently, Bliss defined RT-STING synergy became neutral in all PATECs when repeated in the insert-separated PATEC (Fig. 4C). These findings demonstrate that the cytotoxic efficacy of STING agonist-based RT-IO is highly dependent on direct immune-tumor contact, consistent with engagement of contact-dependent effector pathways such as immune synapse formation, directed degranulation, or death receptor mediated interactions.

### RT-induced cytotoxicity is comparable in TuC and PATEC at 72 h

Next, we evaluated whether RT-induced killing also requires immune cells. We compared RT responses in tumor monocultures (TuC) versus autologous cocultures (PATEC). RT alone significantly increased tumor cell death compared to the control (*p* < 0.001), however responses were indistinguishable between TuC and PATEC conditions (linear mixed-effects model with patient random intercept; Fig. 4D, Additional file 2: Fig. S4C). This suggests that, unlike with the STING agonist, the effect of RT is predominantly tumor-intrinsic within the 72 h assay window in PATEC.

### Immune cell contact alone does not promote spontaneous killing

**Fig. 5:**
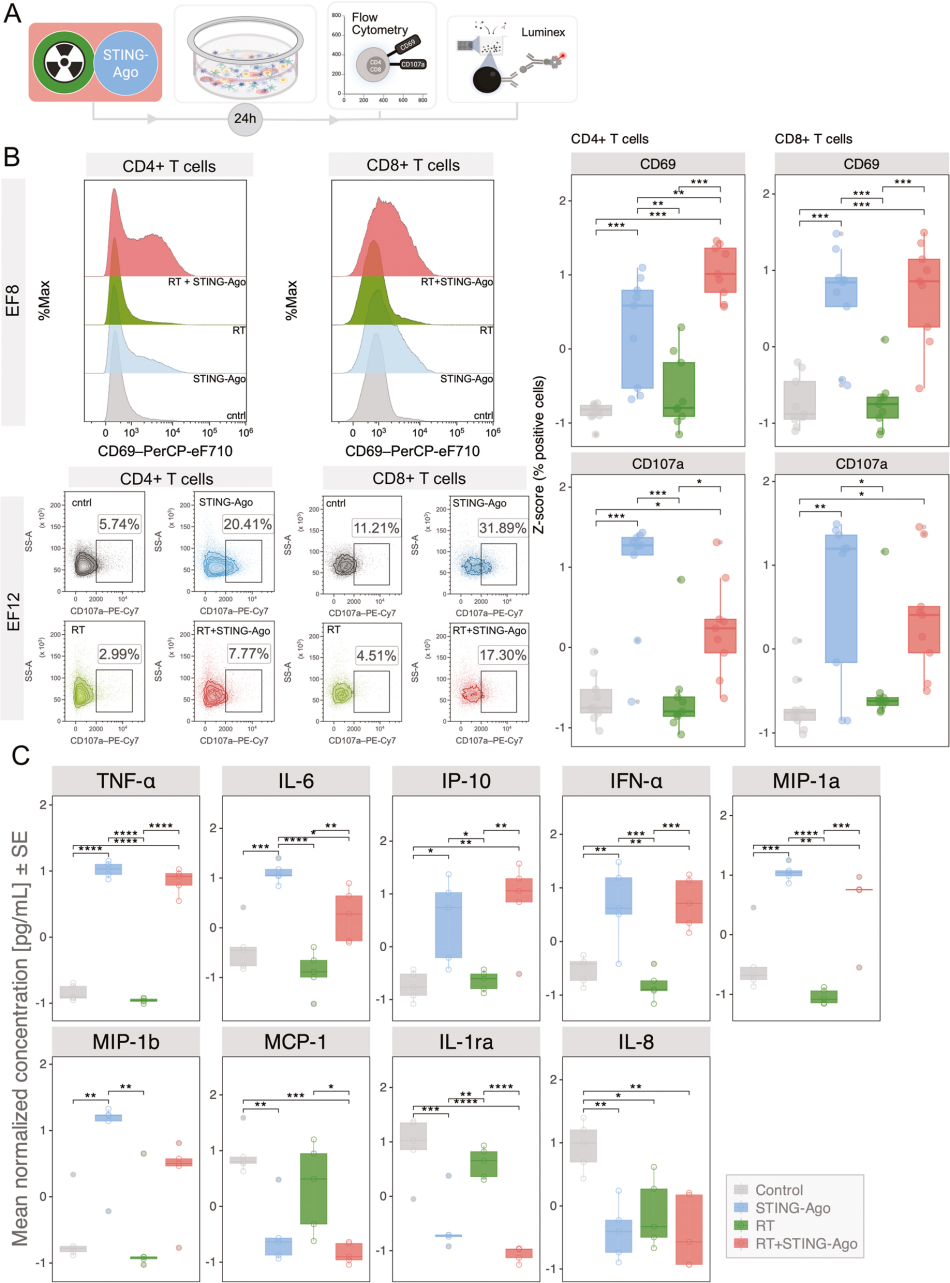
T-cell activation and cytokine induction in effusion cultures following STING agonism and RT. **A** Experimental workflow. **B** CD8⁺ and CD4⁺ T-cell activation (CD69) and degranulation (CD107a) across treatments (*n* = 9 effusions). Example CD69 overlay histograms and CD107a density plots. **C** Cytokines and chemokines in the supernatants after 24 h of treatment (*n* = 5). Treatments: STING agonist (10 µM ADU-S100), RT (8 Gy), RT + STING agonist (8 Gy + 10 µM ADU-S100). **B** Values are normalized to each matched control of the effusion. **C** Values are z-score normalized concentrations. **B**,** C** Boxes show the median and IQR; whiskers 1.5×IQR; dots are the means of technical replicates. Two-way ANOVA; Tukey’s multiple comparisons. *p < 0.05; **p < 0.01; ***p < 0.001; ****p < 0.0001; ns, not significant

To determine whether autologous immune cells exert any basal cytotoxicity in the absence of therapeutic stimulation, we compared untreated tumor cell monocultures (TuC) with direct cocultures (PATEC) and insert-separated PATEC (PATEC (well insert)). Baseline tumor cell death was indistinguishable across all three configurations (linear mixed-effects model; ns), indicating that effusion-derived immune cells do not spontaneously kill tumor cells ex vivo, even when in direct physical contact (Fig. 4E, Additional file 2: Fig. S4D). In summary, these data show that immune cell activity in PATEC requires therapeutic augmentation and that the tumoricidal effects of STING agonist-based RT-IO derive from treatment-induced, not constitutive, immune engagement. This provided the rationale to examine whether early immune activation and cytokine responses accompany the induction of cytotoxicity (Fig. 5).

### T cell activation in malignant effusion cultures following STING agonism and RT

To assess early immune activation following treatment, malignant effusion cultures were exposed to the STING agonist, RT, or combination for 24 h (Fig. 5A). FCM analysis revealed distinct activation and degranulation profiles across T cell subsets. Expression of the activation marker CD69 was highest after combined RT + STING agonist, most prominently in CD4⁺ T cells, whereas CD8⁺ T cells showed a comparable magnitude of CD69 induction under STING agonist alone and the combination (two-way ANOVA with Tukey; *p* < 0.05; Fig. 5B). In contrast, the degranulation marker CD107a was most strongly increased after STING agonist monotherapy in both CD4⁺ and CD8⁺ T cells, while combination treatment produced more moderate induction across patients’ malignant effusions.

These findings suggest that STING agonism promotes degranulation responses, whereas the addition of RT enhances CD69 upregulation on CD4⁺ T cells, indicating distinct but complementary patterns of T-cell activation within the effusion microenvironment at 24 h.

### Pro-inflammatory cytokine and IFN-I responses accompany T cell activation

To explore whether cytokine release increases upon treatment, multiplex profiling of culture supernatants was performed after 24 h (*n* = 5). Combined RT + STING agonist treatment induced a broad pro-inflammatory cytokine response, marked by increased levels of TNF-α, IL-6, IP-10 (CXCL10), IFN-α, MIP-1α (CCL3), and MIP-1β (CCL4) (two-way ANOVA with Tukey; *p* < 0.05; Fig. 5C). In contrast, IFN-β, IL-8, MCP-1, RANTES, IFN-γ, and IL-10 showed no consistent modulation at this early time point (Additional file 2: Fig. S5). This cytokine profile is characteristic of an early IFN-I-driven and chemokine-rich milieu, consistent with STING pathway activation and recruitment of effector leukocytes.

**Fig. 6:**
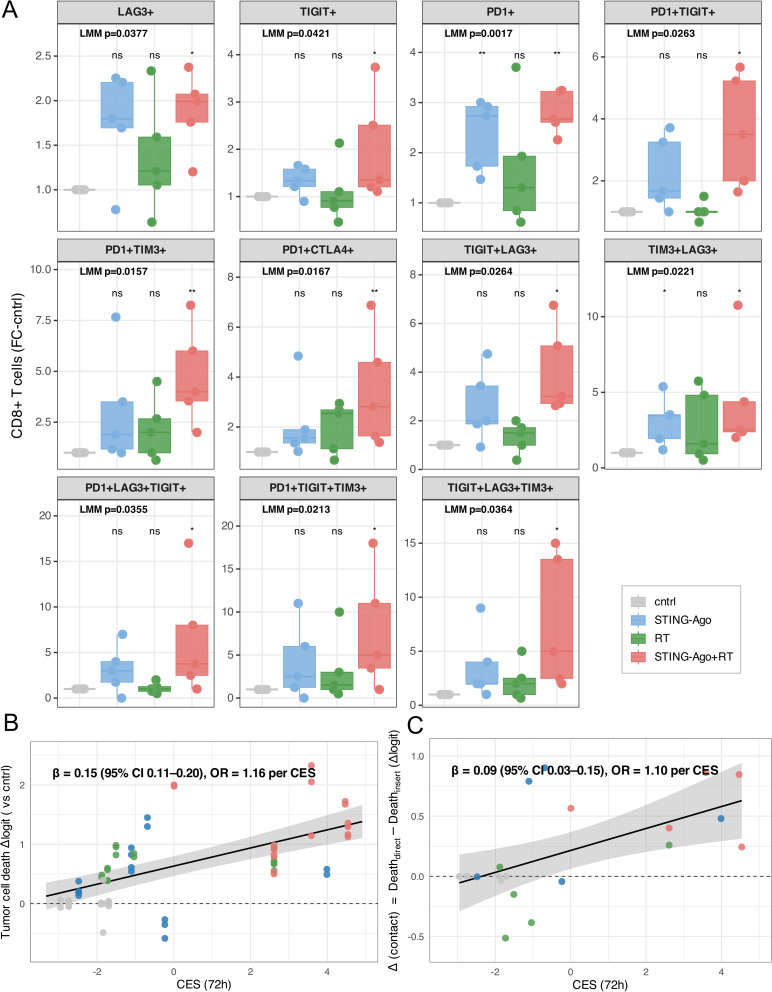
STING agonist and RT induce CD8⁺ checkpoint upregulation and associate with immune cell contact-dependent cytotoxicity. **A** CD8⁺ checkpoint expression in PATEC after 72 h of treatment (cntrl, STING agonist, RT, RT + STING agonist) (*n* = 5 PATECs). Shows fold change of % CD8⁺ cells positive for the indicated checkpoints relative to matched cntrl. Boxes show median and IQR; whiskers 1.5×IQR; dots are individual experiments. LMM on logit-transformed data; patient random intercept; Dunnett contrasts versus cntrl. *p < 0.05; **p < 0.01; ***p < 0.001; ns, not significant. **B** Scatterplot of the association between CD8⁺ checkpoint co-expression score (CES) and overall tumor cell death at 72 h (*n* = 6 PATECs). CES plotted against Δlogit tumor cell death (change in logit-transformed % dead tumor cells vs. cntrl). **C** Scatterplot of the association between CES and the contact-dependent component of tumor cell killing at 72 h (*n* = 5 PATECs). Cell contact-dependent component is defined as Δ(contact) = Δlogit(Direct) − Δlogit(Insert). CES is plotted against Δ(contact). Treatments: STING agonist (10 µM ADU-S100), RT (8 Gy), RT + STING agonist (8 Gy + 10 µM ADU-S100). **B**,** C** Each dot represents one experiment; colors indicate treatment. The black line shows the LMM regression fit and the grey band indicates the 95% confidence interval

Overall, these results show that STING agonism elicits degranulation-dominant T-cell responses, whereas the addition of RT is associated with stronger CD69 upregulation on CD4⁺ T cells, together with a coordinated IFN-I cytokine response at 24 h. This early immunostimulatory phase prompted us to investigate whether sustained checkpoint upregulation and functional engagement of CD8⁺ T cells correlated with tumor cell killing at a later time point (Fig. 6).

### STING-based RT-IO enhances checkpoint co-expression on CD8⁺ T cells

Having established that STING agonism and RT modulate 24 h T cell activation and cytokine release (Fig. 5), we next examined whether prolonged stimulation elicited adaptive checkpoint engagement on cytotoxic T cells. After 72 h of treatment, PATEC cultures exposed to the STING agonist + RT displayed broad upregulation of inhibitory and activation receptors on CD8⁺ T cells (Fig. 6A). Single positive subsets (PD-1⁺, TIGIT⁺, LAG-3⁺) increased relative to matched controls, and multiple combinatorial checkpoint co-expression phenotypes were also enriched following RT + STING agonist (linear mixed-effects models with Dunnett contrasts; *p* < 0.05). The simultaneous induction of multiple checkpoints under this regimen suggests a sustained antigenic stimulation program typical of recently activated effector cells rather than fixed exhaustion.

### Composite checkpoint expression correlates with tumor cell killing

To quantify checkpoint co-expression, we derived a checkpoint co-expression score (CES) that integrates z-normalized frequencies of triple-positive subsets weighted by quadruple-positive enrichment. Across PATECs and treatments, higher CES was correlated with greater tumor cell death at 72 h (Δlogit vs. matched control; Fig. 6B). In a Gaussian mixed-effects model with the patient as a random intercept, the slope was β = 0.15 (95% CI 0.11–0.20), corresponding to an odds ratio (OR) of 1.16 per CES unit (*p* = 1.8 × 10⁻⁸; R²ₘarg = 0.37, R²cond = 0.41). Experiments from RT + STING agonist tended to be in the upper CES-cytotoxicity range, however the fitted relationship was consistent across all conditions, indicating that the degree of checkpoint co-expression quantitatively mirrors the magnitude of cytotoxic efficacy irrespective of treatment type.

### CES is preferentially associated with the contact-dependent component of tumor cell killing

Having established that STING agonist-induced cytotoxicity contains a substantial contact-dependent component (Fig. 4), we next examined whether the CES cytotoxicity relationship was primarily attributable to this contact-mediated fraction. To isolate this effect, we defined Δ(contact) as the difference in treatment-induced cytotoxicity between direct coculture and insert-separated conditions: Δ(contact) = Δlogit(Direct) − Δlogit(Insert). Across PATECs, Δ(contact) increased with CES, with a mixed-effects slope of β = 0.09 (95% CI 0.03–0.15), corresponding to a 10% increase in the odds of contact-dependent killing per CES unit (*p* = 0.0088; R²ₘₐ_r_g = 0.31; Fig. 6C). Importantly, this association persisted after subtraction of contact independent cytotoxicity, indicating that CD8⁺ T cells with higher checkpoint expression preferentially arise under conditions of direct immune-tumor interaction, consistent with an activation program linked to sustained antigen engagement.

The distribution of checkpoint-positive phenotypes is consistent with established CD8⁺ T cell differentiation trajectories. PD-1⁺TIGIT⁺ subsets are characteristic of recently stimulated, antigen-experienced effector populations that retain proliferative and cytotoxic potential, whereas the additional expression of LAG-3 or TIM-3 marks progressively constrained states associated with more sustained antigen exposure. At this early 72 h time point, the positive association between CES and tumor cell killing suggests that multi-checkpoint expression reflects an actively engaged effector program operating under inducible inhibitory feedback, rather than terminal dysfunction.

To explore whether induction of individual checkpoints associated with the benefit of having the corresponding checkpoint inhibitor in the PATEC, we used a quadruple RT-IO screen to correlate the upregulation of PD-1⁺, PD-L1/PD-1⁺, TIGIT⁺, and CTLA-4⁺ on T cells after STING agonist + RT with the additional tumor cell death observed when pembrolizumab, atezolizumab, tiragolumab, or ipilimumab were in culture. In CD8⁺ T cells, higher induction of PD-1, PD-L1/PD-1, and TIGIT tended to coincide with greater incremental killing, whereas analogous associations for CD4⁺ T cells were weaker and more variable; none reached statistical significance in this small cohort, and these analyses were therefore considered exploratory (Additional file 2: Fig. S6).

These hypothesis-generating data suggest that multi-checkpoint expression on CD8⁺ T cells is associated with the magnitude of tumor cell killing, and that this association persists after isolating the contact-dependent component of cytotoxicity. Accordingly, higher CES values capture CD8⁺ states that coincide with conditions in which contact-mediated cytotoxicity constitutes a larger share of the overall response.

## Discussion

In this study, we developed PATEC as a human ex vivo platform to evaluate RT-IO regimens in an immunocompetent TIME. In a combinatorial screen, we found that regimens combining RT with STING agonism were the most tumoricidal in this cohort. Furthermore, PATEC showed STING-dependent cytotoxicity, which was largely mediated by immune cells and was strongly dependent on direct immune-tumor contact. In contrast, within the 72 h PATEC assay window, RT alone appeared to act predominantly through tumor-intrinsic mechanisms. STING-based RT-IO is accompanied by effector T cell activation, an inflammatory/IFN-I associated cytokine milieu, and upregulation of inhibitory checkpoints on CD8⁺ T cells. Collectively, these findings suggest that this platform may help identify and characterize feasible RT-IO combinations.

Within the current landscape of human ex vivo models, PATEC addresses some of the distinct limitations of existing models. While organoid immune cocultures have been used to test ICI, they originate from solid tumors and have a sparse inherent immune infiltrate. They heavily rely on stemcell supporting growth factors, as well as typically requiring extraction, expansion, and reintroduction of immune cells from PBMCs or TILs, rather than utilizing the native autologous TIME [31, 32]. Similarly, tumor-on-a-chip platforms mostly implement immune cell substitutes, require processing of scarce surgical or biopsy material and coincide with high costs owing to microfabrication with a specialized infrastructure [33–37]. In contrast, precision-cut tissue slices and PDTF platforms preserve tissue architecture and stromal context. They are useful for the short-term assessment of checkpoint and cytokine responses but are still limited by restricted source material and a solid tissue interface that does not readily permit systematic separation or repeated perturbation of tumor and immune compartments [38, 39]. To date, malignant effusion-derived cultures have focused on expanding epithelial cells for targeted or chemotherapeutic drug screening, isolating and stimulating T cells for cytotoxicity assays, or testing CAR-T cell cytotoxicity using solely pleural fluid [19, 20, 40–43]. However, the PATEC configuration uses naturally enriched autologous tumor-associated immune cells in the fluid TIME, retaining a plethora of myeloid and lymphoid repertoires of the malignant effusions. Cryopreserved aliquots enable experimental flexibility, spatial compartmentalization allows for the dissection of the responsible cytotoxic effector, and serial sampling offers the potential for longitudinal functional profiling during the course of therapy.

The cellular baseline compositions of the cohorts’ effusions are consistent with previous descriptive characterizations. Across patients there was a pronounced interindividual heterogeneity, with immune compartments variably enriched for macrophages, neutrophils, CD4⁺ and CD8⁺ T cells, NK cells, and B cells, in line with findings from single cell sequencing and multiparametric studies showing diverse immune subsets and patient-specific effusion immune profiles [21, 44, 45]. Although we did not specifically characterize exhaustion, regulatory, or myeloid polarization states at baseline, the effusion-derived immune cells in PATEC did not exhibit spontaneous inherent cytotoxicity. This observation is consistent with mechanistic studies indicating that the effusion TIME constitutes a maladaptive but potentially reversible effector compartment in which CD8⁺ T cells and NK cells are chronically constrained by local factors, yet can regain cytotoxic function upon appropriate stimulation [25, 46–48].

The RT-IO cohort comprised three pancreatic adenocarcinomas, one lung adenocarcinoma, one serous ovarian carcinoma, and one adenocarcinoma of unknown primary, all derived from metastatic effusions. We applied a single 8 Gy fraction as a standardized hypofractionated dose across these heterogeneous histologies, selected on both clinical and immunobiological grounds. Clinically, 8 Gy per fraction falls within established hypofractionated regimens across the represented tumor types: pancreatic SBRT employs fractions of 6–10 Gy [49], radio-immunotherapy regimes for metastatic NSCLC include 3 × 8 Gy [50], ovarian metastases are treated with both conventional palliative and SBRT approaches in this dose range [51], and CUP lacks a disease-specific fractionation standard, with local RT individualized to presentation [52]. Immunobiologically, single fractions in the 6–12 Gy range have been shown to optimally activate cytosolic DNA sensing and type I interferon production via the cGAS-STING pathway, while remaining below the dose threshold at which Trex1-mediated degradation of cytoplasmic DNA attenuates tumor immunogenicity [53, 54].

Within the PATEC system, combinatorial screening showed that RT-IO regimens incorporating innate agonists were consistently among the most effective at inducing tumor cell death, with STING-agonist-containing combinations showing a modest but incrementally more consistent advantage over those including TLR7/8 agonists. These ex-vivo findings are consistent with preclinical models demonstrating that ionizing radiation and STING agonists can be highly efficacious in combination [8, 55–58]. In contrast to these in vivo studies, the PATEC assay assesses local effector activity over a 72 h window and may not capture delayed immune-mediated effects that emerge after the initial RT response, including dendritic cell trafficking, lymph node priming, or abscopal responses, therefore the observed efficacy reflects direct tumor killing within the TIME rather than long-term systemic control [8, 55, 57–59]. In this effector phase, Bliss independence analyses indicated that only a subset of PATECs exhibited a STING-RT synergistic effect, suggesting that patient-specific features of the TIME determine how effectively the combined actions of STING stimulation and irradiation are translated into cytotoxic effector activity. This functional heterogeneity was not restricted to differences between tumor types, as the three pancreatic PATECs showed both synergistic and neutral response classes (Additional file 1: Table S4). However, because downstream RT-IO profiling was limited to six PATECs with small and unbalanced tumor type representation, tumor type effects cannot be disentangled from patient-specific features of the TIME, including the pronounced variability in effusion immune composition described in Fig. 1. Such heterogeneity is consistent with preclinical data demonstrating that tumor-intrinsic cGAS-STING pathway integrity, the mode of STING activation, and its expression level critically modulate responsiveness to RT and STING agonists [60–62]. In this context, the modest and heterogeneous responses observed in early phase trials of intratumoral STING agonists, applied primarily as monotherapy or with PD-1 blockade, are plausibly attributable to variations in STING pathway and to the absence of rationally selected combinations and patient selection strategies [4, 63–65]. These data indicate that effector phase platforms, such as PATEC, could help functionally identify effective innate immunotherapy regimens and nominate patients for further in vivo evaluation.

Comparing PATEC with matched tumor monocultures and transwell separated cocultures enabled us to distinguish tumor-intrinsic from immune-mediated effects of STING-based RT-IO. RT alone produced similar levels of cytotoxicity in primary tumor monocultures and in PATEC, retaining its efficacy when tumor and immune compartments were separated, indicating that RT elicits predominantly DNA-damage-driven tumor-intrinsic cytotoxicity within 72 h in this system [66]. In contrast, the addition of the STING agonist increased tumor cell death only in the presence of immune cells. This benefit was almost completely lost under transwell separation, implicating that STING-dependent cytotoxicity in PATEC is largely indirect and strongly dependent on close immune-tumor apposition. This is consistent with canonical contact-dependent effector mechanisms, including the formation of a cytotoxic immunological synapse, polarized/directed degranulation of lytic granules, and death receptor engagement or release of short-range soluble cytotoxic mediators by cytotoxic lymphocytes and activated myeloid cells [67–70]. Untreated PATEC co‑cultures did not exhibit spontaneous tumor cell killing, even when tumor and immune cells were in close contact. Similarly, human data shows that effusion‑resident NK and CD8⁺ T cells are chronically constrained by the effusion milieu yet can reacquire proliferative and cytotoxic function when removed from suppressive fluid or exposed to appropriate cytokine or antigenic stimulation [25, 46, 47, 71, 72]. These converging lines of evidence support a model in which malignant effusions constitute an immunosuppressive effector niche that remains functionally reversible. In this context, these observations point to an interplay in which RT induces tumor-intrinsic DNA-damage and immunogenic antigen release, whereas STING agonism primarily boosts effusion resident cytotoxic lymphocyte and macrophage function.

An important observation supporting an immune-compartment mechanism was the strong RT + STING agonist synergy in EF2, although STING was undetectable in the matched tumor-cell culture. Unlike EF3 and EF6, EF2 showed essentially no tumor killing with STING agonist monotherapy, indicating that STING activation alone was insufficient in this sample. A plausible explanation is that RT rendered EF2 tumor cells more permissive to contact-dependent immune killing through radiation-induced immunogenic modulation, including enhanced antigen processing and presentation, cell-surface calreticulin exposure, and increased susceptibility to cytotoxic effector engagement [73–75]. RT may additionally have increased the availability of tumor-derived DNΑ and cGAMP for sensing by STING-competent myeloid cells, even when tumor-cell STING was low [55, 76]. In addition to the near-complete loss of effect under transwell separation, these findings are most consistent with a model in which RT primes the tumor target, whereas STING agonism activates the immune effector compartment.

In PATEC, STING agonism and RT modulated T cell activation, checkpoint expression, and the secreted cytokine profile. After 24 h, treatment with the STING agonist alone was sufficient to drive T-cell degranulation. The combination of RT and the STING agonist most strongly increased CD69 expression on CD4^+^ T cells and induced a pro-inflammatory IFN-I cytokine and chemokine profile marked by higher TNF-α, IL-6, CXCL10/IP-10, IFN-α, CCL3, and CCL4. This pattern is concordant with preclinical STING-RT studies, in which IFN-I, TNF-α, and CXCL9/10-rich milieus were induced to support effector activation, as well as with human coculture studies showing that pharmacological STING activation can directly augment the degranulation and cytotoxic function of T cells and NK cells [58, 77–81]. Consistent with sustained antigenic stimulation, the STING agonist and RT induced the most pronounced significant upregulation of PD-1, TIGIT, LAG-3, and TIM-3 on CD8⁺ T cells, which was associated with cytotoxicity. Although high expression of PD-1, LAG-3, TIGIT, and TIM-3 is often interpreted as an exhaustion signature, experimental and clinical data indicate that these receptors are induced sequentially along differentiation and chronic stimulation trajectories. They frequently mark tumor-experienced T cell populations, whose functional state ranges from active to terminally exhausted, depending on the context and timing [82, 83]. In line with data showing that CD8⁺ T cells expressing multiple inhibitory receptors can remain polyfunctional, mediating antitumor activity in patients as well as in humanized models, and that gene signatures enriched for PD-1, LAG-3, TIM-3, and TIGIT expression are associated with clinical benefit from ICI, our data support a similar interpretation in PATEC [84–87]. The positive association between CES and cytotoxicity in PATEC supports the interpretation of CES-high CD8⁺ T-cell states in this early window as engaged effector populations under inducible inhibitory feedback, rather than terminally dysfunctional cells.

The addition of PD-1, PD-L1, CTLA-4, or TIGIT blockade to STING- or TLR7/8-based RT-IO in the quadruple therapy experiment did not produce a significant, consistent further increase in tumor cell death across PATECs. Exploratory analyses suggested that effusions with a stronger induction of the corresponding checkpoint on CD8⁺ T cells under STING + RT tended to derive modest incremental killing from the blockade of that pathway, particularly for TIGIT and PD-L1. These findings are hypothesis-generating but are in accordance with reports that CD8⁺ T cells co-expressing multiple inhibitory receptors can remain functionally competent and enriched for checkpoint-responsive tumor reactivity. They are also consistent with ex vivo “tumor avatar” studies in which PD-1/CTLA-4/TIGIT blockade predominantly modulated progenitor exhausted compartments, clonal composition, and cytokine programs over several days, rather than yielding significant gains in short-term cytotoxicity [84–91].

Although the PATEC model has screening-platform potential, several limitations should be considered. The successful generation of primary tumor cultures for PATECs was modest at 20% and was derived from pan-cancer, as well as diverse pretreatments. Consistent with the pan-cancer sampling, long-term PTCC establishment among cultures attempted varied across tumor types (Additional file 1: Table S3A), with exploratory differences by stage at diagnosis, effusion site, and pancreatic versus non-pancreatic primaries (Additional file 1: Table S3B). Because downstream functional RT-IO profiling was restricted to the six cases that yielded long-term PTCCs (> P3) and corresponding PATECs (Additional file 1: Table S4), the current dataset should be interpreted as proof-of-concept and does not support firm conclusions regarding tumor type specific functional responsiveness. The inherent fact, that there is a finite amount of biological material obtainable from malignant effusions, restricts the complexity and number of follow-up experiments that can be performed. The model’s fluid interface is a double-edged sword; while providing favorable culture conditions it lacks stromal architecture, vasculature, antigen priming environment and pharmacokinetics. Processes dependent on these, such as recruitment of leukocytes, clonal replacement, stromal immunosuppression or dose-limiting toxicities are not recapitulated. The functional behavior of the culture might also not reflect the in vivo response of the cells. Further work is required to establish the clinical representativeness of the culture model.

## Conclusion

PATEC may provide a patient-derived ex vivo platform to functionally test new immunotherapeutic and RT-IO combinations in an autologous, immunocompetent TIME. In this proof-of-concept study, combinations of RT with a STING agonist were the most tumoricidal regimens, with Bliss-defined synergy observed in a subset. STING-dependent cytotoxicity was largely immune-mediated and strongly dependent on direct immune-tumor contact. STING-based RT-IO showed rapid T cell activation, an IFN-I-driven pro-inflammatory cytokine milieu, and upregulated checkpoints on CD8⁺ T cells, which were associated with tumor cell killing. These findings position malignant effusion-derived PATECs as an effector phase platform to rationally develop RT-IO regimens, dissect patient-specific synergy, and generate functional hypotheses for immunotherapy-related biomarkers. Prospective integration of PATEC into precision oncology and early phase trials could help link patient-specific ex vivo responses to clinical outcomes and support more rational selection of immunotherapeutic combinations for patients with advanced solid tumors.

## Supplementary Information

Supplementary Material 1.

Supplementary Material 2.

## Data Availability

The datasets used and analyzed during the current study are available from the corresponding author on reasonable request.

## Abbreviations

RT-IO: Radiotherapy-immunotherapy
RT: Radiotherapy
IO: Immunotherapy
ICI: Immune checkpoint inhibition/inhibitor
PATEC: Patient‑derived autologous tumor-immune effusion co‑culture
PTCC: Primary tumor cell culture
ME: Malignant effusion
TIME: Tumor-immune microenvironment
TME: Tumor microenvironment
TuC: Tumor cell monoculture
STING: Stimulator of interferon genes
STING‑Ago: STING agonist
TLR7/8: Toll‑like receptor 7/8
CTLA‑4: Cytotoxic T‑lymphocyte associated protein 4
PD‑1: Programmed cell death protein 1
PD‑L1: Programmed death‑ligand 1
LAG‑3: Lymphocyte activation gene 3
TIGIT: T cell immunoreceptor with Ig and ITIM domains
IFN‑I: Type I interferons
IFN‑α: Interferon alpha
IFN‑β: Interferon beta
IFN‑γ: Interferon gamma
TNF‑α: Tumor necrosis factor alpha
IL: Interleukin
IL‑1ra: Interleukin‑1 receptor antagonist
IL‑6: Interleukin 6
IL‑8: Interleukin 8
IL‑10: Interleukin 10
IP‑10: Interferon‑gamma-induced protein 10 (CXCL10)
MCP‑1: Monocyte chemoattractant protein 1 (CCL2)
MIP‑1α: Macrophage inflammatory protein 1 alpha (CCL3)
MIP‑1β: Macrophage inflammatory protein 1 beta (CCL4)
RANTES: Regulated on activation, normal T cell expressed and secreted (CCL5)
PBMC: Peripheral blood mononuclear cell
TIL: Tumor infiltrating lymphocyte
NK cell: Natural killer cell
NKT cell: Natural killer T cell
IHC: Immunohistochemistry
FCM: Flow cytometry
FBS: Fetal bovine serum
PBS: Phosphate buffered saline
FC: Fold change
CES: Checkpoint co‑expression score
LMM: Linear mixed-effects model
ANOVA: Analysis of variance
IQR: Interquartile range
CI: Confidence interval
OR: Odds ratio
FDR: False discovery rate
DAB: 3,3’-diaminobenzidine
OD: Optical density
Gy: Gray
DMEM/F12: Dulbecco’s Modified Eagle Medium/Ham’s F‑12 nutrient mixture
