# Supplementary material for: Autologous tumor-immune effusion cocultures enable ex vivo functional profiling of radiotherapy-immunotherapy combinations
Source: J Exp Clin Cancer Res. 2026 Apr 14;45:129. doi: 10.1186/s13046-026-03707-5 (PMC13235057; doi:10.1186/s13046-026-03707-5)
Supplement: Supplementary file 1 — Supplementary Material 1. [file 13046_2026_3707_MOESM1_ESM.docx]

**Table S1A Key reagents used for sample processing and cell culture**

| **Reagent type** | **Designation** | **Source** | **Identifier** |
| --- | --- | --- | --- |
| Kit | EasySep™ Human CD45 Depletion Kit | STEMCELL Technologies | Cat#17898 |
| Dye | Zombie Violet™ Fixable Viability Kit | BioLegend | Cat#423114 |
| Dye | Zombie Yellow™ Fixable Viability Kit | BioLegend | Cat#423104 |
| Beads | UltraComp eBeads™ | Invitrogen | Cat#01-2222-42 |
| Reagent | Accutase® | Sigma | Cat#A6964 |
| Reagent | ACK lysis buffer | Gibco | Cat#A1049201 |
| Reagent | CryoStor® CS10 | STEMCELL | Cat#C2874 |
| Small molecule | Y-27632 dihydrochloride | MedChemExpress | Cat#HY-10071 |

**Table S1B Antibodies used for immunohistochemistry**

| Reagent type | Designation | Source | Identifier(s) | Additional information |
| --- | --- | --- | --- | --- |
| Antibody | Anti-EpCAM (clone HEA125) | Miltenyi Biotec | Cat#130-113-262; RRID:AB_2726063 | IHC 1:80 |
| Antibody | Anti-pan Cytokeratin (clone C11) | Cell Signaling Technology | Cat#4545; RRID:AB_490860 | IHC 1:500 |
| Antibody | Anti-STING/TMEM173 (clone D2P2F) | Cell Signaling Technology | Cat#13647; RRID:AB_2732796 | IHC 1:400 |

**Table S1C Antibodies used for flow cytometry immune phenotyping**

| **Reagent type** | **Designation** | **Source** | **Identifier(s)** | **Additional information** |
| --- | --- | --- | --- | --- |
| Antibody | Anti-CD45 | BioLegend | Cat#368514; RRID:AB_2566374 | Clone 2D1; AF700 |
| Antibody | Anti-CD45 | Invitrogen | Cat# 48-9459-42;  RRID:AB_1603240 | Clone 2D1; eFluor450 |
| Antibody | Anti-EpCAM | Invitrogen (eBioscience) | Cat#53-8326-42; RRID:AB_11219279 | Clone MH99; AF488 |
| Antibody | Anti-CD3 | BioLegend | Cat#317306; RRID:AB_571907 | Clone OKT3; FITC |
| Antibody | Anti-CD4 | BioLegend | Cat#317416; RRID:AB_571945 | Clone OKT4; APC |
| Antibody | Anti-CD8a | BioLegend | Cat#300908 (PE); RRID:AB_314112 \| Cat#344724 (AF700); RRID:AB_2562790 | Clone HIT8A (PE) / SK1 (AF700); PE and/or AF700 |
| Antibody | Anti-CD56 | BioLegend | Cat#318306; RRID:AB_604101 | Clone HCD56; PE |
| Antibody | Anti-CD14 | BioLegend | Cat#301814; RRID:AB_389353 | Clone M5/E2; PE-Cy7 |
| Antibody | Anti-CD11c | Invitrogen (eBioscience) | Cat#46-0116-42; RRID:AB_10596368 | Clone 3.9; PerCP-eFluor710 |
| Antibody | Anti-HLA-DR | BioLegend | Cat#307618; RRID:AB_493586 | Clone L243; APC-Cy7 |
| Antibody | Anti-CD20 | BioLegend | Cat#302310; RRID:AB_314258 | Clone 2H7; APC |
| Antibody | Anti-CD163 | Invitrogen (eBioscience) | Cat#46-1639-42; RRID:AB_2573722 | Clone GHI/61; PerCP-eFluor710 |
| Antibody | Anti-CD66b | BioLegend | Cat#305106; RRID:AB_2077857 | Clone G10F5; PE |
| Antibody | Anti-CD11b | BioLegend | Cat#101226; RRID:AB_830642 | Clone M1/70; APC-Cy7 |
| Antibody | Anti-CD86 | BioLegend | Cat#305428; RRID:AB_2563823 | Clone IT2.2; BV650 |
| Antibody | Anti-CD107a | BioLegend | Cat#328618; RRID:AB_11147955 | Clone H4A3; PE-Cy7 |
| Antibody | Anti-CD69 | Invitrogen (eBioscience) | Cat#46-0699-42; RRID:AB_2573694 | Clone FN50; PerCP-eFluor710 |
| Antibody | Anti-CTLA-4 | BioLegend | Cat#349922; RRID:AB_2566198 | Clone L3D10; PE-Dazzle 594 |
| Antibody | Anti-TIGIT | Invitrogen (eBioscience) | Cat#46-9500-42; RRID:AB_10853679 | Clone MBSA43; PerCP-eFluor710 |
| Antibody | Anti-LAG3 | BioLegend | Cat#369310; RRID:AB_2629753 | Clone 11C3C65; PE-Cy7 |
| Antibody | Anti-PD-1 | BioLegend | Cat#329932; RRID:AB_2562256 | Clone EH12.2H7; BV510 |
| Antibody | Anti-PD-L1 | BioLegend | Cat#329736; RRID:AB_2629582 | Clone 29E.2A3; BV785 |
| Antibody | Anti-TIM3 | BioLegend | Cat#345026; RRID:AB_2565717 | Clone F38-2E2; APC-Cy7 |

**Table S1D Isotype control antibodies used for flow cytometry**

| **Reagent type** | **Designation** | **Source** | **Identifier** |
| --- | --- | --- | --- |
| Isotype control | IgG1 κ | BioLegend; Thermo Fisher Scientific (eBioscience) | Cat#400144; RRID:AB_2923250 (AF700) \| Cat#400112; RRID:AB_2847829 (PE) \| Cat#400125; RRID:AB_2861433 (PE-Cy7) \| Cat#400176; RRID:AB_2923261 (PE/Dazzle 594) \| Cat#400171; RRID:AB_2714004 (BV510) \| Cat#46-4714-80; RRID:AB_1834454 (PerCP-eFluor710) |
| Isotype control | IgG2a κ | Thermo Fisher Scientific (eBioscience) | Cat#11-4724-42; RRID:AB_1963642 (FITC) \| Cat#12-4724-42; RRID:AB_1603322 (PE) |
| Isotype control | IgG2b κ | BioLegend | Cat#400356; RRID:AB_3097678 (BV785) |
| Isotype control | IgG mouse/rat/etc. | Thermo Fisher Scientific (eBioscience) | Cat#17-4321-81; RRID:AB_470181 (Rat IgG2a κ, APC) |

**Table S2 Sample characteristics and culture outcomes of malignant effusions (n = 29)**

| **EF** | **Site** | **Primary** | **Histol** | **Stage** | **Metastatic at sampling** | **Grade** | **Sex** | **Age** | **Cells (×10⁶)** | **Cult** | **PTCC>3** | **RT** | **SysTx** | **Cx** | **TT** | **ICI** | **ET** | **PATEC** | **T-cell** |
| --- | --- | --- | --- | --- | --- | --- | --- | --- | --- | --- | --- | --- | --- | --- | --- | --- | --- | --- | --- |
| EF1 | A | Pancreas | Adeno | IV | Y | G3 | F | 57 | 366 | Y | Y | N | N | N | N | N | N | Y | N |
| EF2 | A | Pancreas | Adeno | IV | Y | U | M | 67 | 2400 | Y | Y | N | N | N | N | N | N | Y | N |
| EF3 | A | CUP | Adeno | IV | Y | U | M | 52 | 463 | Y | Y | N | N | N | N | N | N | Y | N |
| EF4 | P | Lung | Adeno | IV | Y | G2 | M | 70 | 442 | Y | Y | Y | Y | N | Y | N | N | Y | N |
| EF5 | P | Pancreas | Adeno | IV | Y | G3 | M | 62 | 6300 | Y | Y | N | Y | Y | N | N | N | Y | N |
| EF6 | P | Ovary | Serous OC | IV | Y | G3 | F | 48 | 155 | Y | Y | N | Y | Y | Y | N | N | Y | N |
| EF7 | P | Breast | Breast ca | I | Y | G3 | F | 37 | 300 | Y | N | Y | Y | Y | Y | N | Y | N | Y |
| EF8 | P | Breast | Breast ca | I | Y | G2 | F | 65 | 45 | N | NA | Y | N | N | N | N | N | N | Y |
| EF9 | P | Gastric | Adeno | IV | Y | G3 | F | 55 | 426 | Y | N | N | Y | Y | Y | N | N | N | Y |
| EF10 | P | Ovary | Serous OC | III | Y | G3 | F | 41 | 550 | Y | N | Y | Y | Y | Y | N | N | N | Y |
| EF11 | P | Ovary | Serous OC | IV | Y | G3 | F | 57 | 1360 | Y | N | N | N | N | N | N | N | N | Y |
| EF12 | P | Pancreas | Adeno | II | Y | G3 | M | 51 | 800 | Y | N | N | Y | Y | N | N | N | N | Y |
| EF13 | P | Pancreas | Adeno | IV | Y | G3 | F | 81 | 80 | N | NA | N | Y | Y | N | N | N | N | Y |
| EF14 | P | Ovary | Serous OC | III | Y | G1 | F | 49 | 94 | N | NA | N | Y | Y | Y | N | N | N | Y |
| EF15 | P | Appendix | Adeno | IV | Y | G3 | F | 45 | 40 | N | NA | N | Y | Y | Y | N | N | N | Y |
| EF16 | A | Colorectal | Adeno | III | Y | G3 | F | 68 | 12 | N | NA | N | Y | Y | Y | N | N | N | N |
| EF17 | P | Lung | Adeno | IV | Y | U | F | 74 | 253 | Y | N | N | N | N | N | N | N | N | N |
| EF18 | A | Colorectal | Adeno | IV | Y | G2 | M | 55 | 129 | Y | N | Y | Y | Y | N | N | N | N | N |
| EF19 | P | Gastric | Adeno | III | Y | G3 | M | 58 | 1903 | Y | N | Y | Y | Y | Y | N | N | N | N |
| EF20 | P | Gastric | Adeno | III | Y | G3 | M | 58 | 123 | Y | N | N | Y | Y | Y | N | N | N | N |
| EF21 | P | Ovary | Serous OC | I | Y | G1 | F | 63 | 750 | Y | N | N | Y | Y | Y | N | N | N | N |
| EF22 | P | Lung | Adeno | III | Y | G3 | M | 63 | 160 | Y | N | Y | Y | Y | Y | Y | N | N | N |
| EF23 | P | Breast | Breast ca | II | Y | G3 | F | 38 | 75 | N | NA | N | Y | Y | Y | Y | N | N | N |
| EF24 | P | Breast | Breast ca | II | Y | G3 | F | 75 | 99 | N | NA | Y | Y | Y | Y | N | Y | N | N |
| EF25 | A | Pancreas | Adeno | I | Y | G2 | M | 72 | 22 | N | NA | N | Y | Y | N | N | N | N | N |
| EF26 | P | Lung | Adeno | IV | Y | U | F | 66 | 2704 | Y | N | Y | Y | Y | N | Y | N | N | N |
| EF27 | P | Lung | NEC | IV | Y | G3 | M | 77 | 600 | Y | N | Y | Y | Y | N | Y | N | N | N |
| EF28 | P | Lung | Adeno | III | Y | G2 | M | 65 | 1480 | Y | N | N | N | N | N | N | N | N | N |
| EF29 | P | Lung | Adeno | III | Y | G2 | F | 63 | 55 | N | NA | Y | Y | Y | Y | N | N | N | N |

Site: A, ascites; P, pleural effusion. Sex: F, female; M, male. Binary variables are coded Y/N.

Cells (×10⁶) denotes the total nucleated cell yield at processing. Cult indicates whether PTCC cultivation was attempted and was performed only when total nucleated cell yield at processing was ≥100 × 10⁶ cells; samples below this threshold were not attempted.

PTCC>3 indicates long-term PTCC establishment, defined as proliferation beyond three passages (>P3). NA in PTCC>3 denotes not applicable (culture not attempted). Unknown (U) denotes missing clinical information. Abbreviations: Adeno, adenocarcinoma; Breast ca, breast carcinoma; Serous OC, serous ovarian carcinoma; NEC, neuroendocrine carcinoma; RT, radiotherapy; SysTx, any systemic therapy prior to effusion sampling (modalities not mutually exclusive); Cx, chemotherapy; TT, targeted therapy; ICI, immune checkpoint inhibitor therapy; ET, endocrine therapy; PATEC, patient-derived autologous tumor-immune effusion co-culture; PTCC, primary tumor cell culture; IQR, interquartile range.

**Table S3A PTCC establishment by tumor type among cultures attempted (n = 20)**

| **Primary tumor** | **Established PTCC** |
| --- | --- |
|  | **n/N (%)** |
| Lung | 1/6 (16.7) |
| Pancreas | 3/4 (75.0) |
| Ovary | 1/4 (25.0) |
| Gastric | 0/3 (0.0) |
| Breast | 0/1 (0.0) |
| Colorectal | 0/1 (0.0) |
| CUP | 1/1 (100.0) |

Cultures were attempted only in samples with ≥100 × 10⁶ nucleated cells. Successful PTCC establishment was defined as sustained proliferation beyond three passages. Percentages represent the proportion of established cultures within each tumor type (n established / N attempted).

**Table S3B Exploratory univariable associations with PTCC establishment among cultures attempted (n = 20)**

| **Characteristic** | | | **Established PTCC** | | |
| --- | --- | --- | --- | --- | --- |
|  |  |  | **n/N (%)** | **P value*** | |
| **Effusion site** | | | | | |
| Ascites | | | 3/4 (75.0) | 0.061 | |
| Pleural effusion | | | 3/16 (18.8) |  | |
| **Primary tumor (Pancreas vs non-pancreas)** | | | | | |
| Pancreas | | | 3/4 (75.0) | 0.061 | |
| Non-pancreas | | | 3/16 (18.8) |  | |
| **Stage at diagnosis** | | | | | |
| IV | | | 6/12 (50.0) | 0.042 | |
| I–III | | | 0/8 (0.0) |  | |
| **Radiotherapy prior to sampling** | | | | | |
| Yes | | | 1/8 (12.5) | 0.325 | |
| No | | | 5/12 (41.7) |  | |
| **Any systemic therapy prior to sampling** | | | | | |
| Yes | | | 3/14 (21.4) | 0.303 | |
| No | | | 3/6 (50.0) |  | |
| **Chemotherapy prior to sampling** | | | | | |
| Yes | | | 2/13 (15.4) | 0.122 | |
| No | | | 4/7 (57.1) |  | |
| **Immune checkpoint inhibitor therapy prior to sampling** | | | | | |
| Yes | | | 0/3 (0.0) | 0.521 | |
| No | | | 6/17 (35.3) |  | |
| **Targeted therapy prior to sampling** | | | | | |
| Yes | | | 2/9 (22.2) | 0.642 | |
| No | | | 4/11 (36.4) |  | |
| **Sex** | | | | | |
| Male | | | 4/11 (36.4) | 0.642 | |
| Female | | | 2/9 (22.2) |  | |
|  | Established (n = 6) | Not established (n = 14) | | |  |
|  | median [IQR] | median [IQR] | | | ****** |
| Age | 59.5 [53.3–65.8] | 58.0 [55.0–64.5] | | | 1.00 |
| Total nucleated cell yield at processing (×10⁶) | 452.5 [385.0–1915.8] | 575.1 [264.8–1220.0] | | | 0.779 |

* Two-sided Fisher’s exact test.

** Two-sided Mann-Whitney U test.

Analyses were restricted to cultures attempted (n = 20), defined a priori as samples with ≥100 × 10⁶ nucleated cells at processing. PTCC establishment was defined as proliferation beyond three passages. Analyses are exploratory and descriptive due to small subgroup sizes and correlated predictors.

**Table S4 Selected tumor characteristics and functional outcomes in PATEC assays**

| **EF ID** | **Effusion site** | **Primary tumor** | **Histology** | **TCD - FC vs control (STING)** | **TCD - FC vs control (RT)** | **TCD - FC vs control (RT + STING)** | **Bliss ΔE (%)** | **Synergy classification** |
| --- | --- | --- | --- | --- | --- | --- | --- | --- |
| EF1 | Ascites | Pancreas | Adenocarcinoma | 1.68 | 1.25 | 1.79 | 0.7 | Neutral |
| EF2 | Ascites | Pancreas | Adenocarcinoma | 0.73 | 1.82 | 2.61 | 31.2 | Synergistic |
| EF3 | Ascites | CUP | Adenocarcinoma | 1.50 | 1.81 | 3.28 | 25.7 | Synergistic |
| EF4 | Pleural effusion | Lung | Adenocarcinoma | 1.58 | 1.37 | 1.99 | 6.0 | Neutral |
| EF5 | Pleural effusion | Pancreas | Adenocarcinoma | 1.12 | 1.33 | 1.32 | -6.6 | Neutral |
| EF6 | Pleural effusion | Ovary | Serous ovarian carcinoma | 1.04 | 0.97 | 1.15 | 13.8 | Synergistic |

FC, fold change of % Zombie Violet™⁺ (dead) tumor cells relative to matched untreated control at 72 h. FC values represent mean fold-changes across replicate measurements per effusion and treatment. Bliss ΔE, Bliss synergy index (percentage points) for RT + STING. Abbreviations: TCD, tumor cell death; RT, radiotherapy; STING, stimulator of interferon genes agonist; EF, effusion ID; CUP, cancer of unknown primary.
