# Supplementary material for: Autologous tumor-immune effusion cocultures enable ex vivo functional profiling of radiotherapy-immunotherapy combinations
Source: J Exp Clin Cancer Res. 2026 Apr 14;45:129. doi: 10.1186/s13046-026-03707-5 (PMC13235057; doi:10.1186/s13046-026-03707-5)
Supplement: Supplementary file 2 — Supplementary Material 2. [file 13046_2026_3707_MOESM2_ESM.docx]

**Fig. S1 Cellular composition of effusions used for T cell activation.** Stacked bar charts show the proportion of tumor cells versus total CD45⁺ leukocytes, each bar scaled to 100% (n=9). The adjacent bars illustrate the immune subsets within the CD45⁺ compartment, up to 100%.


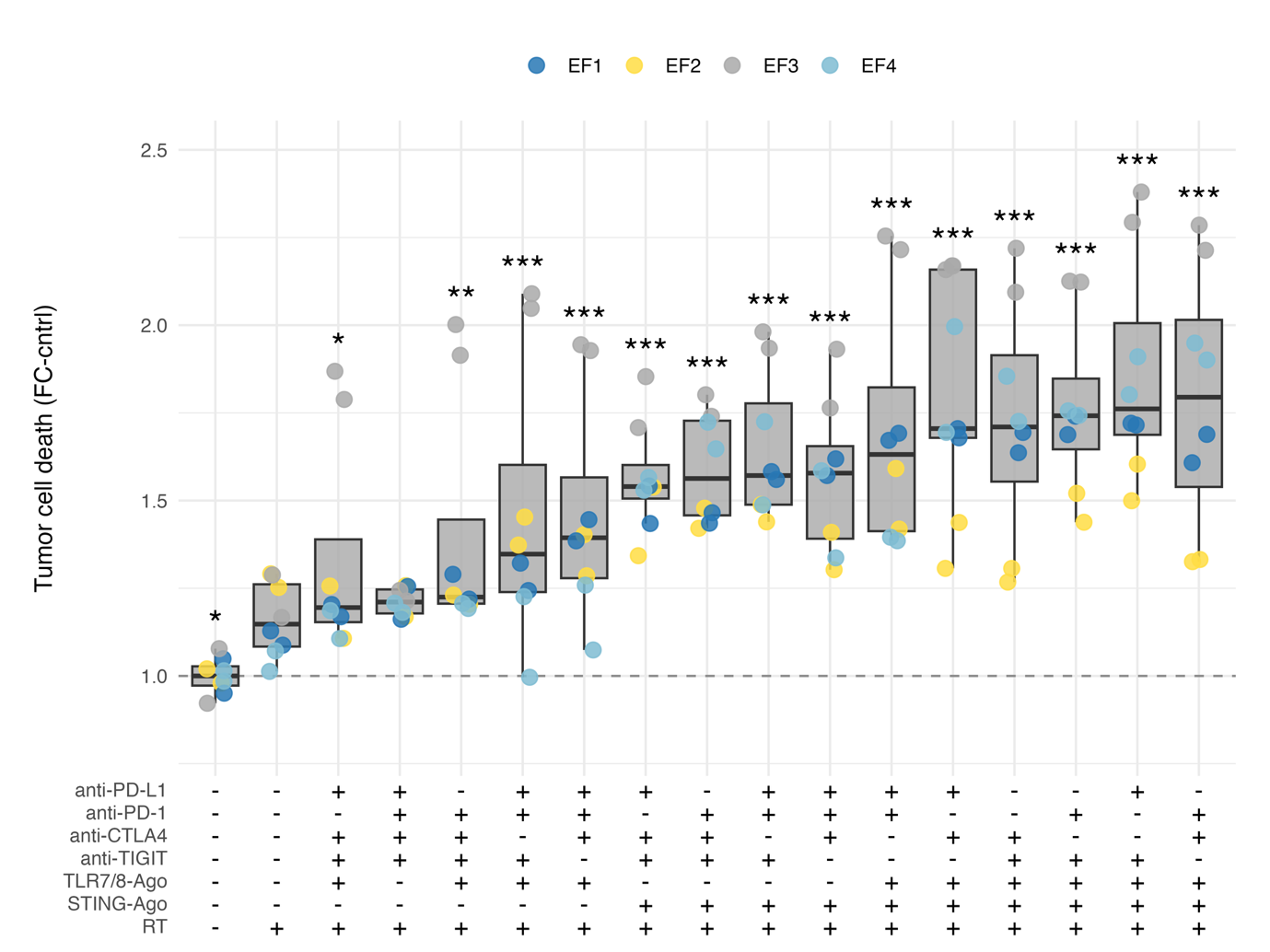
 **Fig. S2A Quadruple RT–IO screening shows differential efficacies in PATEC.** Boxplots show tumor cell death (% Zombie Violet™+ tumor cells; fold change relative to matched cntrl (TuCntrl)) in PATEC (EF1–EF4, n = 4) after 72 h of treatment. Each box represents one unique RT–IO regimen. The matrix below the plot indicates which components were included (+) or absent (−) for each combination. Colored dots denote technical replicates. Boxes show median and IQR; whiskers 1.5×IQR. LMM; patient random intercept and replicate set nested within patient; Holm‑adjusted contrasts comparing each combination to RT alone (8 Gy). *p < 0.05; **p < 0.01; ***p < 0.001.

**Fig. S2B Component-wise effects of RT–IO screening in PATEC.** Boxplots show tumor cell death (fold change) comparing conditions in which the indicated immunotherapeutic component was absent (–) versus present (+) across the RT–IO screen in PATEC (EF1–EF4, n = 4). Colored dots denote technical replicates. Boxes show median and IQR; whiskers 1.5×IQR. Effect of component inclusion (present vs absent) from a multivariable LMM (FoldChange ~ CTLA4 + STING + TLR7/8 + PD L1 + PD 1 + TIGIT + RT + (1|patient)); Holm. *p < 0.05; **p < 0.01; ***p < 0.001.


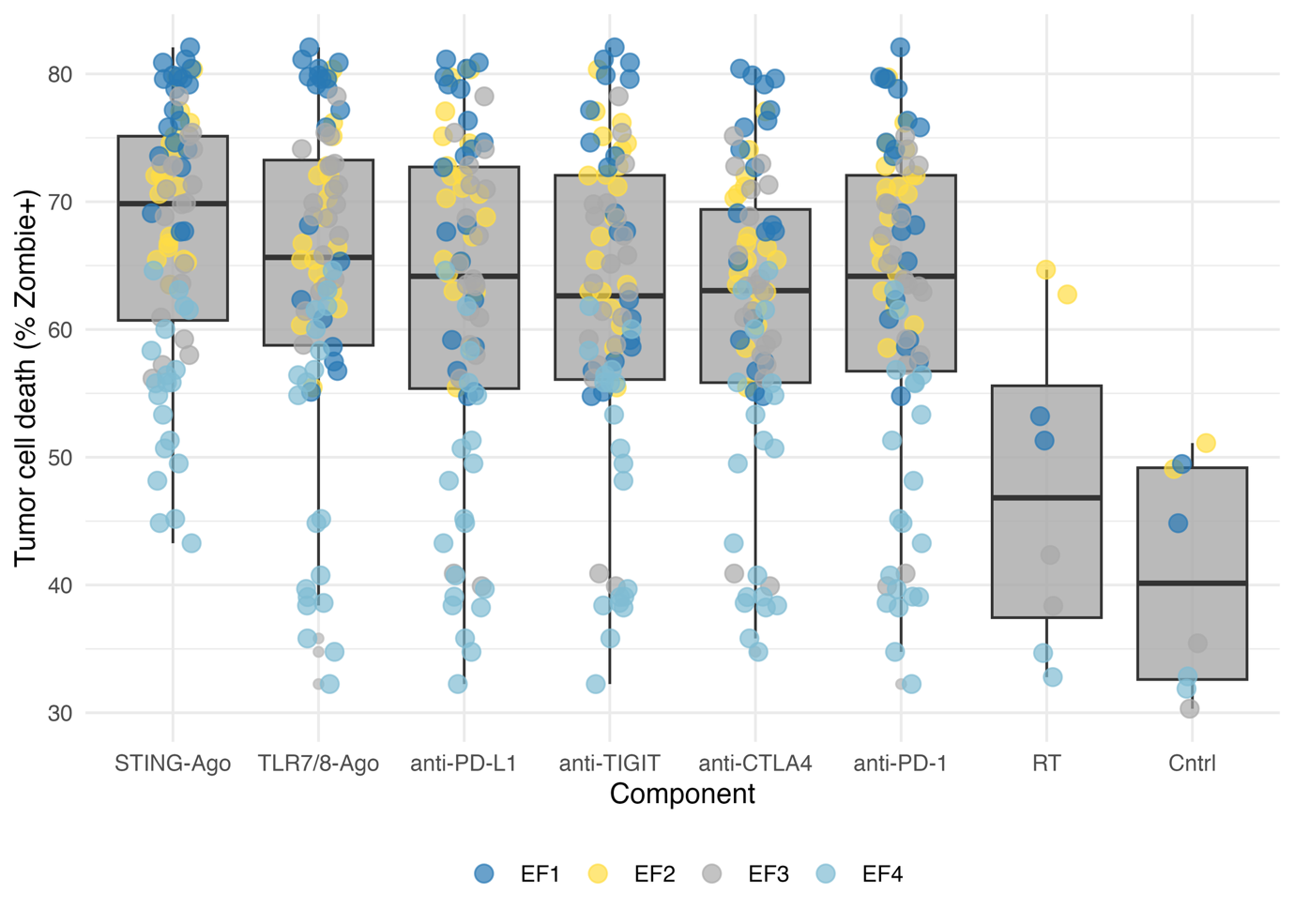
 **Fig. S2C Raw tumor cell death values across the quadruple RT–IO screen in PATEC.** Boxplots show tumor cell death (% Zombie Violet™⁺ tumor cells) across the quadruple RT–IO screen in PATEC (EF1–EF4, n = 4), grouped by the indicated treatment component. Colored dots denote technical replicates, with colors indicating individual PATECs. Boxes indicate median and IQR; whiskers 1.5×IQR.


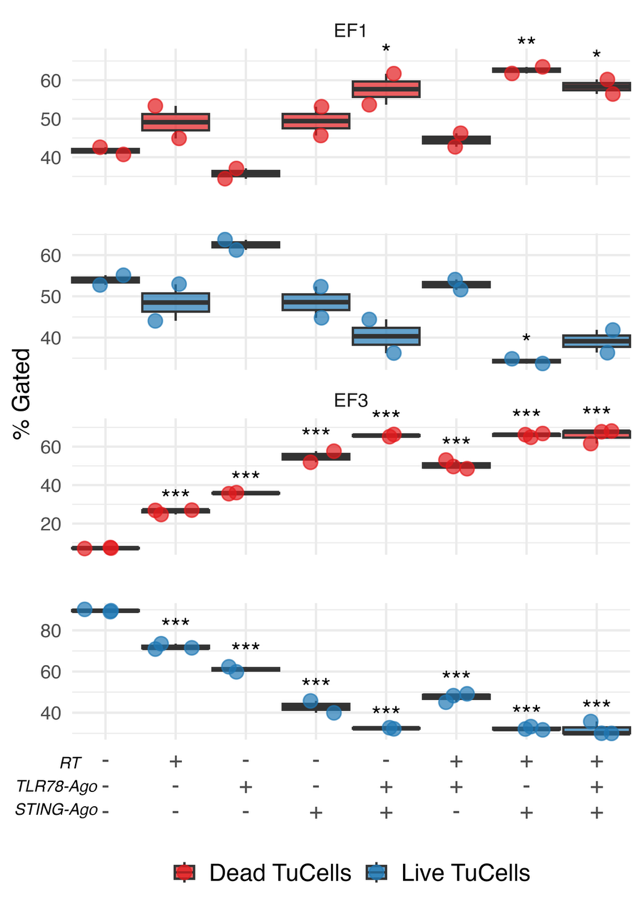


**Fig. S2D Raw live and dead tumor cell fractions in representative PATECs treated with STING agonist, TLR7/8 agonist and RT-based combinations.** Representative PATECs EF1 and EF3 were treated for 72 h with RT, STING-Ago, TLR7/8-Ago, or their combinations. Red boxplots show dead tumor cells and blue boxplots show live tumor cells, quantified by flow cytometry as % gated tumor cells. Dots indicate technical replicates. Boxes indicate median and IQR; whiskers 1.5×IQR.

**
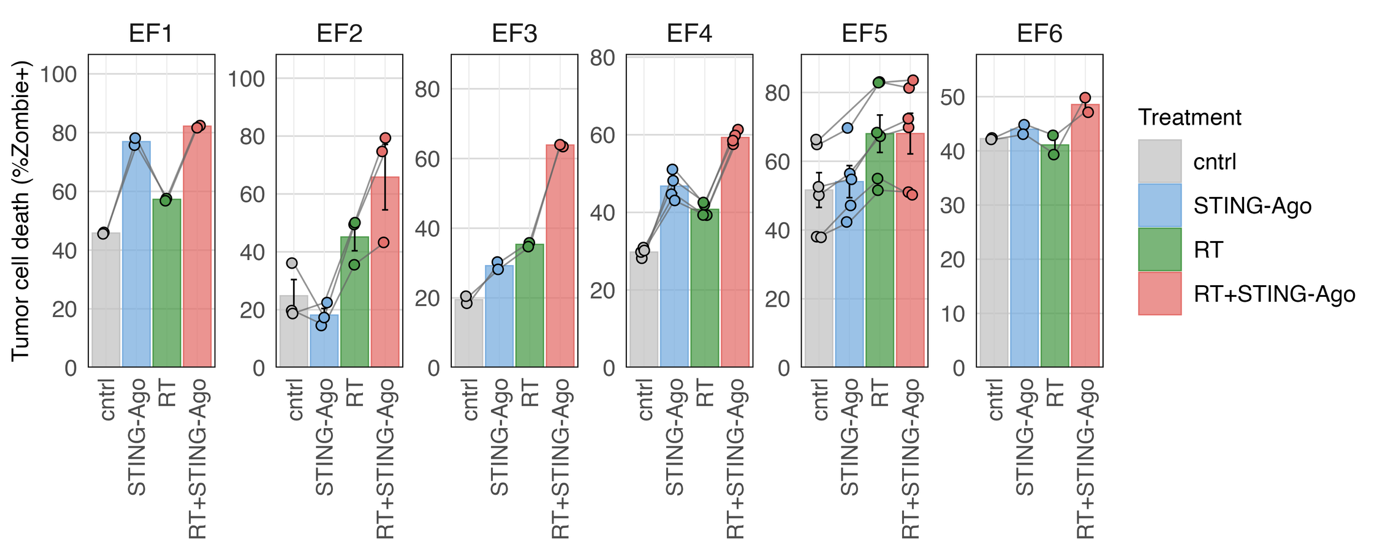
**

**Fig. S3A Raw tumor cell death values in individual PATECs treated with STING agonist and RT.** Bar plots show tumor cell death (% Zombie Violet™⁺ tumor cells) in PATECs EF1–EF6 after 72 h of treatment with cntrl, STING-Ago, RT, or RT + STING-Ago. Bars indicate mean values across biological replicates, and dots indicate individual replicates.

**
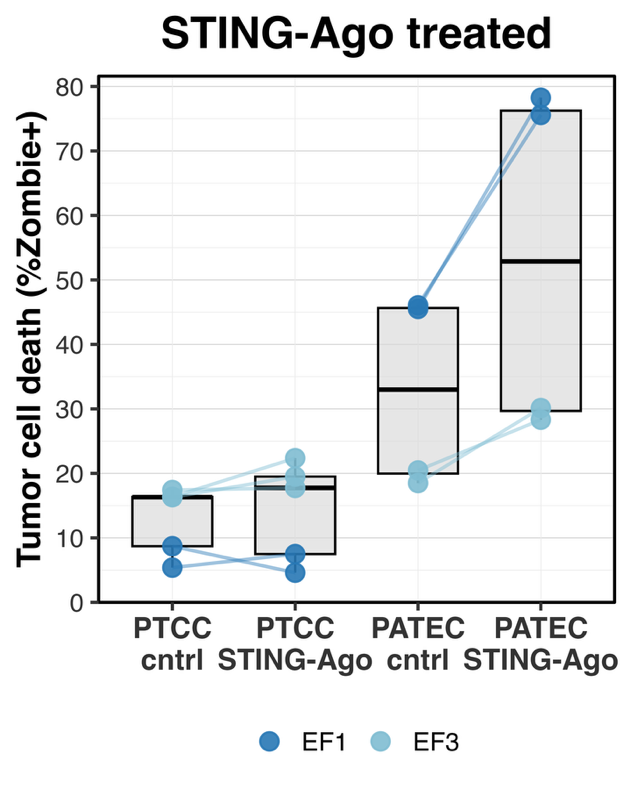
**

**Fig. S3B Raw tumor cell death values in matched PTCC and PATEC after STING agonist treatment.** Boxplots show tumor cell death (% Zombie Violet™⁺ tumor cells) at 72 h in primary tumor monocultures (PTCC) and matched autologous co-cultures (PATEC) under cntrl and STING-Ago conditions in EF1 and EF3. Colored dots denote individual replicates and lines connect matched samples. Boxes indicate median and IQR; whiskers 1.5×IQR.


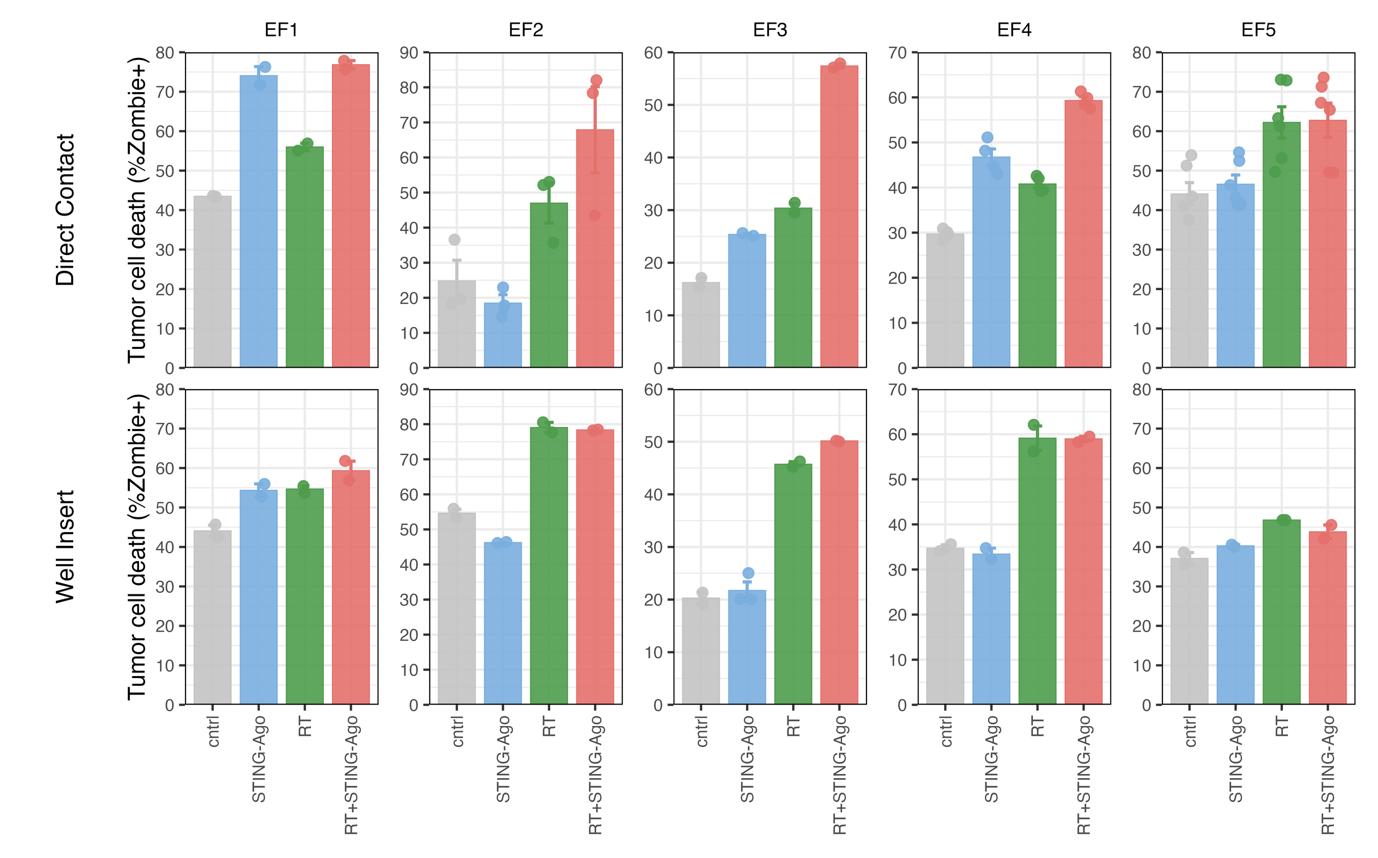


**Fig. S4A Raw tumor cell death values in direct and insert-separated PATECs across treatment conditions.** Bar plots show tumor cell death (% Zombie Violet™⁺ tumor cells) in PATECs EF1–EF5 after 72 h under direct co-culture or well insert separation with cntrl, STING-Ago, RT, or RT + STING-Ago. Bars indicate mean values across replicates and dots indicate individual technical replicates.


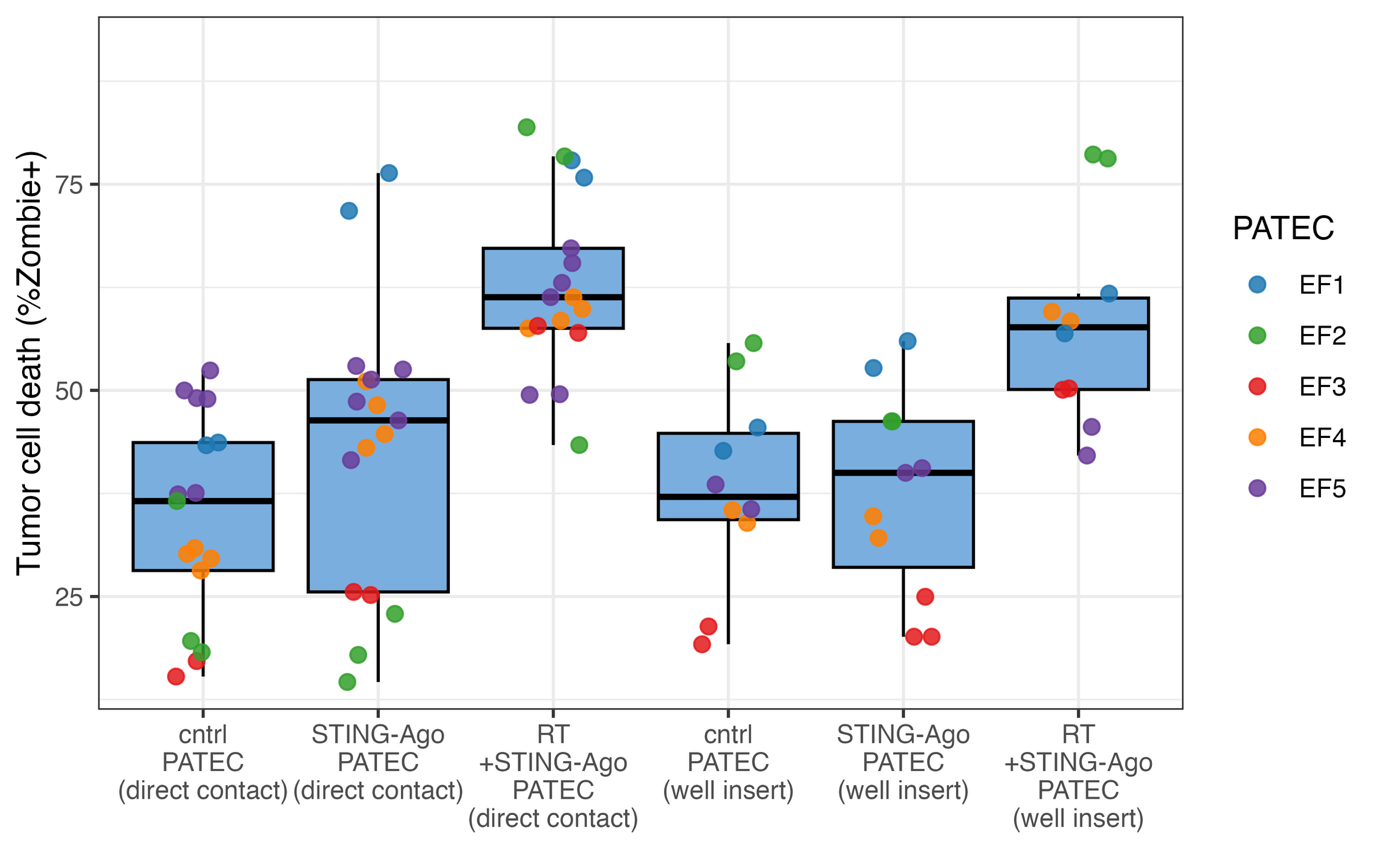


**Fig. S4B Pooled raw tumor cell death values for direct versus insert-separated PATECs.** Boxplots show tumor cell death (% Zombie Violet™⁺ tumor cells) across PATECs EF1–EF5 at 72 h for cntrl, STING-Ago, and RT + STING-Ago under direct contact and well insert conditions. Colored dots indicate individual PATECs. Boxes indicate median and IQR; whiskers 1.5×IQR.


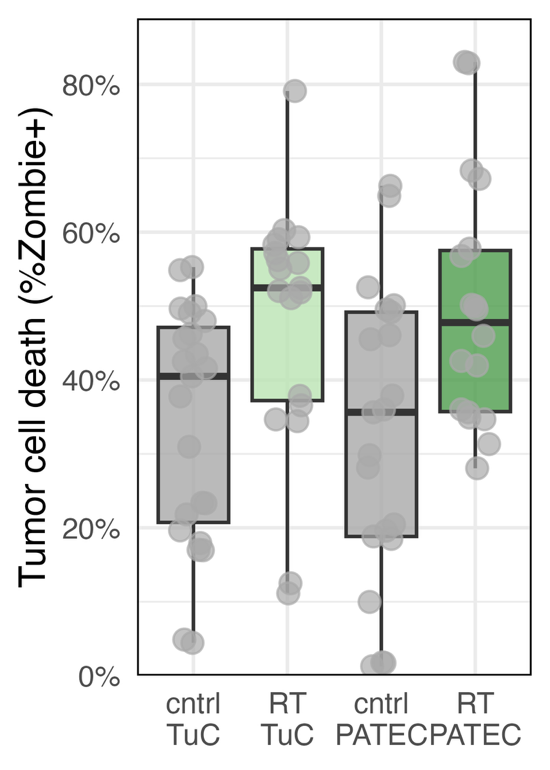


**Fig. S4C Raw tumor cell death values in tumor monocultures and co-cultures after RT.** Boxplots show tumor cell death (% Zombie Violet™⁺ tumor cells) at 72 h in untreated and RT-treated tumor cell monocultures (TuC) and matched direct PATECs (n = 5). Dots indicate replicates. Boxes indicate median and IQR; whiskers 1.5×IQR.

**Fig. S4D Baseline tumor cell death in untreated tumor monocultures and co-culture configurations.** Boxplots show tumor cell death (% Zombie Violet™⁺ tumor cells) at 72 h in untreated TuC, direct PATEC, and insert-separated PATEC (well) across EF1–EF6. Colored dots indicate individual PATECs. Boxes indicate median and IQR; whiskers 1.5×IQR.

**Fig. S5 Cytokines without consistent modulation after STING-Ago + RT.** Malignant effusion cultures were treated ex vivo for 24 h with treatments (cntrl, STING-Ago, RT, RT + STING-Ago) and cytokine concentrations in supernatants were quantified using a Luminex® multiplex assay (n = 5 effusions). Boxplots show z-score-normalized mean concentrations for RANTES, IFN-β, IFN-γ and IL-10 across treatments. Boxes indicate median and IQR; whiskers 1.5×IQR; dots are means of replicates per effusion. Treatments: STING-Ago (10 µM ADU-S100), RT (8 Gy), RT + STING-Ago (8 Gy + 10 µM ADU-S100). Two-way ANOVA; Tukey’s multiple comparisons.

**Fig. S6 Correlation between checkpoint induction after STING-Ago + RT and tumor cell death after STING-Ago + RT + CPI in CD8⁺ (left) and CD4⁺ (right) T cells.** Heatmaps show Pearson correlation coefficients (r) between STING-Ago + RT –induced checkpoint expression and Δ tumor cell death for STING-Ago + RT + ICI in PATEC by adding the corresponding checkpoint inhibitor to STING-Ago + RT in the quadruple RT-IO experiment (EF1–EF4, n = 4 PATECs). For each antibody target pair (PD-1–Pembrolizumab, PD-L1/PD-1–Atezolizumab, TIGIT–Tiragolumab, CTLA-4–Ipilimumab), Δ tumor cell death is defined as the difference in tumor death fold change between wells containing that antibody and wells lacking it which definitely have treatments STING-Ago + RT. Checkpoint induction is quantified as log₂ fold change in the frequency of the corresponding checkpoint-positive CD8⁺ (left) or CD4⁺ (right) T cell subset at Day 3 in PATEC (STING-Ago+RT vs cntrl). Tile colors encode the strength and direction of Pearson r (red, positive; blue, negative; scale −1 to 1) and each tile is annotated with r and the corresponding P value (Holm-adjusted across checkpoints).
